# Cross-Reactive SARS-CoV-2 Neutralizing Antibodies From Deep Mining of Early Patient Responses

**DOI:** 10.3389/fimmu.2021.678570

**Published:** 2021-06-15

**Authors:** Georgia Bullen, Jacob D. Galson, Gareth Hall, Pedro Villar, Lien Moreels, Line Ledsgaard, Giada Mattiuzzo, Emma M. Bentley, Edward W. Masters, David Tang, Sophie Millett, Danielle Tongue, Richard Brown, Ioannis Diamantopoulos, Kothai Parthiban, Claire Tebbutt, Rachael Leah, Krishna Chaitanya, Sandra Ergueta-Carballo, Deividas Pazeraitis, Sachin B. Surade, Omodele Ashiru, Lucia Crippa, Richard Cowan, Matthew W. Bowler, Jamie I. Campbell, Wing-Yiu Jason Lee, Mark D. Carr, David Matthews, Paul Pfeffer, Simon E. Hufton, Kovilen Sawmynaden, Jane Osbourn, John McCafferty, Aneesh Karatt-Vellatt

**Affiliations:** ^1^ IONTAS Ltd., Cambridge, United Kingdom; ^2^ Alchemab Therapeutics Ltd., London, United Kingdom; ^3^ Leicester Institute of Structural and Chemical Biology and Department of Molecular and Cell Biology, University of Leicester, Leicester, United Kingdom; ^4^ National Institute for Biological Standards and Control, Potters Bar, United Kingdom; ^5^ LifeArc, Stevenage, United Kingdom; ^6^ Abcam, Cambridge, United Kingdom; ^7^ European Molecular Biology Laboratory, Grenoble, France; ^8^ Barts and The London School of Medicine and Dentistry, Queen Mary University of London, London, United Kingdom

**Keywords:** COVID-19, antibodies, convergence, phage display, SARS-CoV-2 variants

## Abstract

Passive immunization using monoclonal antibodies will play a vital role in the fight against COVID-19. The recent emergence of viral variants with reduced sensitivity to some current antibodies and vaccines highlights the importance of broad cross-reactivity. This study describes deep-mining of the antibody repertoires of hospitalized COVID-19 patients using phage display technology and B cell receptor (BCR) repertoire sequencing to isolate neutralizing antibodies and gain insights into the early antibody response. This comprehensive discovery approach has yielded a panel of potent neutralizing antibodies which bind distinct viral epitopes including epitopes conserved in SARS-CoV-1. Structural determination of a non-ACE2 receptor blocking antibody reveals a previously undescribed binding epitope, which is unlikely to be affected by the mutations in any of the recently reported major viral variants including B.1.1.7 (from the UK), B.1.351 (from South Africa) and B.1.1.28 (from Brazil). Finally, by combining sequences of the RBD binding and neutralizing antibodies with the B cell receptor repertoire sequencing, we also describe a highly convergent early antibody response. Similar IgM-derived sequences occur within this study group and also within patient responses described by multiple independent studies published previously.

## Introduction

In the past two decades, three major virus outbreaks caused by coronaviruses have emerged. The latest, COVID-19 caused by Severe Acute Respiratory Syndrome Coronavirus 2 (SARS-CoV-2), has resulted in a pandemic with over 153 million people infected and causing over 3.2 million deaths. The majority of drug development efforts have been focused on vaccine development, with 93 and 184 programs going through clinical and preclinical evaluation, respectively, at the beginning of May 2021 (Source: WHO). Despite the accelerated development timeframes and regulatory approval of the leading vaccine candidates, the world is still one or two years away from attaining population immunity due to the manufacturing and logistical challenges of mass vaccinating billions of people. Therefore, monoclonal antibodies have potential as a key component in the early fight against COVID-19. Viral neutralizing antibodies can offer a two-in-one approach, being used both to treat symptomatic individuals following acute exposure, and as a prophylactic to protect healthcare workers and at-risk groups, including individuals who respond poorly to vaccines.

There are 25 experimental anti-SARS-CoV-2 monoclonal antibody treatments undergoing clinical trials. This includes two combination products, bamlanivimab plus etesevimab and casirivimab plus imdevimab, which received Emergency Use Approval from the Food and Drug Administration (FDA) for the treatment of patients with mild to moderate COVID-19. These two antibody treatments have shown to reduce hospitalization and deaths by 70% in non-hospitalized COVID-19 patients in phase III clinical trials. Most well-characterized and highly potent neutralizing antibodies in both clinical and preclinical development (including the antibodies mentioned above) target the SARS-CoV-2 S protein and its receptor binding domain (RBD) (1). The majority of these neutralizing antibodies have been derived from single cell screening of memory B cells from COVID-19 patients in their convalescent phase of disease (blood samples were collected on average 32 days after the onset of symptoms, **Table S1**).

**Figure 1:**
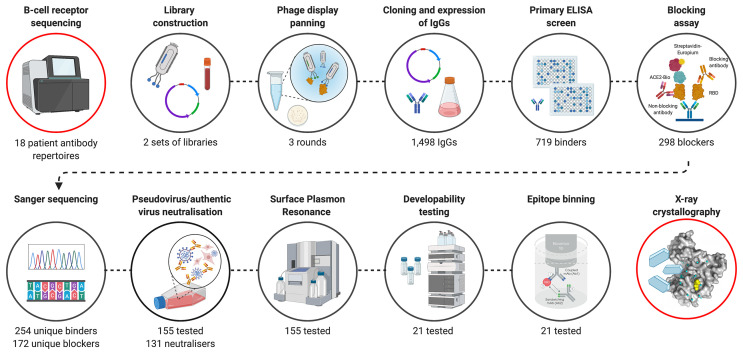
Overview of SARS-CoV-2 antibody discovery and analysis of patient response to COVID-19​. Antibody genes isolated from the PBMC’s of 18 COVID-19 patients were used to for B cell receptor repertoire sequencing and to construct phage display libraries. Characterization of phage display derived antibodies using high throughput expression, primary binding assay and biochemical ACE-2 receptor blocking assays and DNA sequencing resulted in testing of 155 unique antibodies for pseudovirus neutralization and surface plasmon resonance. A final panel of 21 antibodies were subjected to authentic virus neutralization, epitope binding and developability assessment. ​Finally, structures of a complementary pair of antibodies with two different mechanism of viral neutralization in complex with RBD were determined using X-ray crystallography. The V_H_ sequences of RBD binding and pseudoviral neutralizing antibodies were co-clustered with whole BCR repertoire sequencing from the patients and published antibodies to understand the nature and dynamics of the early antibody response. Figure was prepared using BioRender.

Here, we present a complementary discovery approach (**Figure 1**), which combines BCR repertoire sequence analysis with functional selection by phage display technology. This approach allowed the isolation of hundreds of anti-SARS-CoV-2 antibodies from the antibody repertoires of patients in the acute phase of disease (blood samples collected on average 11 days (range 4-20) after the onset of symptoms). A comprehensive search of patient-derived phage display libraries combined with high-throughput biochemical and functional screening resulted in the discovery of highly potent neutralizing antibodies, with diverse epitopes and distinct mechanisms of action. An emphasis on developability testing as part of early discovery screening facilitated selection of a panel of well characterized antibodies with biophysical properties de-risked for downstream development and manufacturing. Armed with the knowledge of functional binding activity, antibody sequences discovered by phage display were co-clustered with whole BCR repertoire sequencing from the patients and published antibodies to understand the nature and dynamics of the early antibody response, including the isotype usage, clonal expansion and the level of convergence.

## Materials and Methods

### Recombinant Antigens and Control Antibodies

For expression of the RBD subdomain of the SARS-CoV-1 (residues Arg306 to Phe527) and SARS-CoV-2 (residues Arg319 to Phe541) spike protein and human ACE2, genes encoding the proteins were synthesized and cloned upstream of either an Fc tag or rCD4-Avi tag, both with an additional 6 x His tag in mammalian expression vectors (2). The constructs were expressed in Expi293F™ cells (Thermo Fisher, A14527) and purified by affinity chromatography using Nickel-NTA agarose (Invitrogen, R90115). SARS-CoV-2 S1 protein with a His tag (S1N-C52H3) and SARS-CoV-2 S protein active trimer with His tag (SPN-C52H8) were separately purchased from ACRO biosystems. MERS-CoV S1 with a His tag was purchased from Sino Biological (40069-V08H).

All antigens with an Avi tag were biotinylated enzymatically using BirA biotin-protein ligase (Avidity, Bulk BirA) while non-Avi tagged antigens were biotinylated chemically using EZ-Link Sulfo-NHS-Biotin (Thermo Fisher, A39256). V_H_ and V_L_ sequences of SARS-CoV-2 control antibodies (COV2-2196, COV2-2130, S309, B38, H4, and CR3022) were obtained from the CoV-AbDab database (3) and cloned into pINT3/pINT54 IgG expression vectors (4) as synthetic gene fragments. The antibodies were expressed using the Expi293™ system and purified using Protein-A affinity chromatography (Generon, PC-A100). Control antibody SAD-S35 was purchased from ACRO biosystems.

### Sample Collection and Total RNA Preparation

Peripheral blood was obtained from a subset of 18 patients who were admitted to medical wards at Barts Health NHS Trust, London, UK with acute COVID-19 pneumonia, after informed consent by the direct care team. Blood collection and processing, followed by total RNA preparation, was carried out as previously described (5). RNA was split for use in both construction of phage libraries and BCR sequencing.

### Library Construction

Two library strategies were employed, both using the antibodies in an scFv format with variable heavy chain (V_H_) and variable light chain (V_L_) fragments joined by a flexible linker peptide (Gly**_4 _**Ser Gly_4 _Ser Gly_3 _Ala Ser). The first strategy was to construct hybrid libraries where donor V_H_s were combined with a library of naïve kappa and lambda V_L_s, whereas the second strategy was to construct a complete patient derived library by assembling V_H_s and V_L_s on a donor-by-donor basis. Antibody V_L_ and V_H_ repertoires were obtained through cDNA synthesis from total RNA using First-Strand cDNA synthesis kit (GE Healthcare, 27926101), followed by PCR amplification. The cDNA template for V_H_s were synthesized using an IgG-specific primer (5’- AGTAGTCCTTGACCAGGCAG -3’) and the cDNA template V_L_s were synthesized using a pd(N)_6_ random hexamer primer to allow for amplification of both lambda and kappa sequences. For the hybrid library strategy, the V_H_s from the cDNA template were amplified and cloned into naïve kappa and lambda light chain libraries as described before (6).

For the complete patient derived library approach, the V_H_s and V_L_s (both lambda and kappa) from each donor were amplified and assembled on a donor-by-donor basis. The primary amplification of V_H_s for the hybrid libraries was also used as the first step for the generation of V_H_s of the complete patient derived libraries. Assembled fragments were cloned into the phage display vector pIONTAS1 using NcoI/NotI cloning sites (6). Final libraries were obtained by electroporation of electrocompetent TG1 cells (Lucigen) with the purified ligation products.

### Phage Display Panning, Subcloning and High Throughput IgG Expression

The hybrid and fully patient derived libraries (see Methods) were subjected to three rounds of phage display selections against biotinylated RBD-Fc-His, RBD-rCD4-Avi-His, and S1-His were carried out as previously described (6). The first and second rounds used selection antigen concentrations of 50 nM and 5 nM respectively. In the third round, each selection was performed at two selection antigen concentrations, 0.25 nM and 0.05 nM. Antibody genes were isolated from the third-round selection populations and sub-cloned into the pINT3 and pINT54 IgG1 mammalian expression vectors (4). Transfection-quality plasmid DNA was prepared for 1498 clones in 96 well plate format (Biobasic, BS415). The DNA was then expressed by transient transfection in Expi293F™ cells (Thermo Fisher, A14527) in 96 well plates at 700 µL/well scale.

### Primary Screening, Sequencing

An affinity capture assay was performed as the initial screening of the 1,498 expressed IgGs. The assay was performed as described elsewhere (7) using 1 nM biotinylated RBD-rCD4-His or S1-His. V_H_s and V_L_s were sequenced using Eurofins Genomics and Genewiz Sanger sequencing services. To identify unique combinations of heavy CDR3 and light CDR3, antibody frameworks and CDR regions were annotated and analyzed using Geneious Biologics (Biomatters).

### SARS-CoV-2 RBD-ACE2 Blocking Assay

Black 96-well immune plates (Thermo Scientific, 10030581) were coated with mouse anti-rCD4 antibody (Bio-Rad, MCA1022R) at 4°C overnight. The following day, plates were blocked with 3% (w/v) milk powder (Marvel) in PBS, before incubation with purified recombinant SARS-CoV-2 RBD-rCD4-His (1.25 µg/mL) for 1 hour. To identify antibodies that could block the interaction between RBD and ACE2, the anti-SARS-CoV-2 antibodies were pre-incubated with 2 nM of recombinant ACE2-Fc-His-Biotin for 30 minutes before transferring to the RBD-coated plates. The IgGs and ACE2 were incubated in the plates for 1 hour and ACE2-Fc-Biotin was detected by DELFIA Eu-N1 Streptavidin (Perkin Elmer, 1244-360) using the DELFIA-TRF system (Perkin Elmer).

### Expression and Purification

For medium scale expression for detailed characterization, all antibodies were expressed at 50 mL scale in ExpiCHO or Expi293 system (Thermo Fisher). Expressed antibodies were purified using protein-A affinity chromatography (by HiTrap Fibro PrismA unit, Cytiva, 17549855) followed by size exclusion chromatography (HiLoad Superdex 200 16/600, Cytiva, 28-9893-35).

For single concentration pseudovirus assay, SPR, and cross-reactivity evaluation, the antibodies expressed in 96 well plates (Expi293 cells) were purified by protein-A affinity chromatography (Generon, PC-A100), using 96 well filter plates (Whatman Polystyrene Unifilter Microplates, GE Healthcare, 11313535). After purification, antibodies were buffer exchanged into PBS using Zeba™ Spin Desalting Plates, 7K MWCO (ThermoFisher Scientific, 89807).

### Pseudovirus Neutralization Assay

To generate SARS-CoV-2 lentiviral pseudotyped virus, 5 x 10^6^ HEK293T/17 cells were seeded within a 10 cm dish. The following day, plasmids encoding the HIV-1 *gag-pol* genes (p8.91), a firefly luciferase reporter gene (pCSFLW) and the SARS-CoV-2 Spike gene (pCAGGS SARS-2-Spike) were transfected concurrently at a ratio of 1:1.5:1 µg, respectively, using Fugene-HD (Promega) transfection reagent. After overnight incubation, culture media was replenished. Supernatant was harvested at 48- and 72-hours post-transfection and stored at -80°C.

For the assay, target cells were prepared by transient transfection of HEK293T/17 cells with 2 µg ACE-2 and 150 ng TMPRSS2 expression plasmids using Fugene-HD (Promega) transfection reagent. 24 hours post-transfection cells were detached and seeded at 20,000 cells/well within a 96-well plate and incubated at 37°C and 5% CO_2_ for at least 2 hours. Three-fold serial dilutions of antibodies were prepared within 60 µL DMEM supplemented with 10% FBS and 1% penicillin/streptomycin. To each well, 60 µL containing 200 TCID50 (50% Tissue Culture Infective Dose) of SARS-CoV-2 lentiviral pseudotyped virus was added and incubated for 1 hour at 37°C. Following incubation, 100 µL of the purified antibodies and pseudotyped virus mix was transferred to the target cells and incubated for 72 hours at 37°C and 5% CO_2_. To acquire results, luciferase expression was detected using the Promega Bright-Glo™ assay system and GloMax Navigator plate reader, following manufacturer’s instructions. The half maximal inhibitory concentration (IC_50_) was calculated using GraphPad version 8 as previously described (8).

### Authentic SARS-CoV-2 Virus Neutralization Assay and Combination Testing

VERO CCL-81 cells were seeded at 20,000 cells/well in a 96-well plate in DMEM (Sigma, D6546) supplemented with 2 m**M** L-Glutamine, 10% Fetal bovine serum (PAN Biotech UK) and 1% penicillin or streptomycin. The following day, two-fold serial dilutions of the antibodies in 60 µL of serum free DMEM were added to the same volume of media containing 100 TCID_50_ of SARS-CoV-2 Australia/VIC01/2020 isolate (Centre For AIDS Reagents cat. no. 100980) and incubated for 1 hour at 37°C. The media from the VERO CCL-81 cells was removed and 100 µL of the antibody and virus mix was added to each well. 100 µL of DMEM supplemented with 4% FBS was added to each well. After 48 hours, cells were fixed with PBS containing 4% formaldehyde in PBS, for 1 hour at room temperature. Cells were permeabilized with 0.1% Triton X-100 in PBS for 15 minutes at room temperature. Cells were blocked with 3% milk in PBS containing 0.05% tween-20. Virus infection was detected using an HRP-conjugated anti-N protein antibody (The Native Antigen Company, MAB12184-HRP) for 1 hour at room temperature. Signal was detected using TMB substrate and stopped with H_2_SO_4_. The half maximal inhibitory concentration (IC_50_) was calculated using GraphPad version 8 as previously described (8).

Effect of the antibodies combinations was evaluated in the neutralization assay by combining two antibodies at the ratio 1:1. Two-fold serial dilutions of the antibodies individually or in combinations were added to 100 TCID_50_ of SARS-CoV-2, Australia/VIC01/2020 isolate. Assay was performed as described above. Response to each antibody concentration was normalized to the virus only value (0% neutralization) and cell only value (100% neutralization). The Combination Index (CI) for the antibodies combinations was calculated using CompuSyn (9, 10).

### Affinity Determination Using Surface Plasmon Resonance

Surface Plasmon Resonance (SPR) experiments were performed using the Sierra SPR-32 instrument (Bruker). All measurements were performed at 25°C. A high-capacity amine sensor (Bruker, 1862614) was activated using EDC/NHS, before protein G was added at 150 µg/mL with a flow rate of 15 µL/min for 400 seconds resulting in immobilization of 3,000-5,000 RU per sensor spot. The sensor spots were inactivated using ethanolamine. IgG antibodies at 5 nM concentrations were captured at 15 µL/min for 180 seconds resulting in an average response of 100-200 RU. SARS-COV2 RBD-rCD4 at concentrations ranging from 20 nM to 0.625 nM was injected at 30 µL/min for 120 seconds and dissociation was recorded for 600 seconds. Following dissociation, the sensors were regenerated using 10 mM glycine-HCl, pH 1.5. The initial kinetics were conducted with 2 analyte concentrations used for each antibody. Measurements for the final panel of 21 antibodies were conducted in triplicates and for determination of kinetic constants, at least 4 analyte concentrations were used for each antibody.

### Epitope Binning

The panel of anti-RBD human antibodies was segregated into epitope bins using the classical sandwich Octet format (**Figure S4**) on Anti-Human Fc Capture (AHC) biosensors (Sartorius, 18-5060). The experimental workflow was carried out in cycles as follows: Load of Ab1 (5 minutes; 5 µg/mL), Quench/Block with irrelevant human IgG (5 minutes; 50 µg/mL; Jackson ImmunoResearch, 009-000-003), Wash in PBS (2 minutes), Load of SARS CoV-2 RBD (5 minutes; 5 µg/mL; Acro BioSystems, SPDC52H3), Baseline in PBS (2 minutes), Association of Ab2 (5 µg/mL; 5 minutes), Dissociation in PBS (2 minutes). Before and after each cycle, PBS-hydrated AHC biosensors underwent of 3x5-second cycles of regeneration (10 mM Glycine pH 1; Sigma; G7403) and neutralization (PBS). Reagents (200 µL/well) dispensed into black non-binding 96-well plates (Greiner; 655209) were maintained at 30°C for 5 minutes prior to and throughout the duration of the experimental cycles. Each antibody was investigated in both orientations (i.e. Ab1 *vs* Ab2). Irrelevant human IgG as Ab2 served as a control for reference subtraction. The data was acquired on an Octet HTX instrument using the 16 channels-high sensitivity mode with shaking (1000rpm). Software programs used were Octet Acquisition software (version 11.1) and Octet Data Analysis HT (version 11.1).

### B Cell Receptor Repertoire Sequencing

Three different V_H_ sequence datasets were integrated for analysis: 1) The V_H_ sequence data from the antibodies isolated in the present study using phage display, 2) the B cell receptor repertoire V_H_ sequence data generated from the same patients and previously published (5) and (3) all V_H_ sequences in CoV-AbDab [accessed 28^th^ April 2021] annotated as having a human-derived V gene segment, and having V and J gene segment annotations, and CDRH3 sequence determined.

Each sequence from the combined dataset was processed using IgBlast (11) to determine V and J germline gene segment usage, and locations of the CDRs and FWRs. Mutation count was determined by the number of mismatches between the sequence and its inferred germline using the shazam R package (12). For dataset 2, the isotype of each sequence was possible to determine by comparison to germline constant region sequences. For datasets 1 and 3, this information was not available.

Sequences were clustered into groups using a previously described algorithm (5). This is a greedy clustering algorithm, run with a threshold requiring sequences within a cluster to have the same V and J gene segment, and no more than 1 AA mismatch per 10 AAs in the CDRH3. This threshold has been previously shown to group together sequences that are sufficiently similar to be considered part of the same B cell clonal expansion, and likely targeting the same epitope. The cluster center is defined as the most common sequence within the cluster. All datasets, and all samples within each dataset were clustered together to identify overlap between the datasets.

### Determination of the Structure of Anti-SARS-CoV-2 Antibodies in Complex With RBD

The Fab fragments for anti-SARS-CoV-2 antibodies ION_300 and ION_360 and SARS-CoV-2 receptor binding domain (RBD) were expressed in Expi293F™ cells (Thermo Fisher, A14527). The antibody:RBD complexes were mixed and co-purified by size exclusion chromatography in 20 mM Tris-HCl (pH 7.5) and 50 mM NaCl. For crystallization, antibody:RBD complex samples were concentrated to 5-10 mg/mL.

All crystals were obtained by the vapor diffusion method at 19oC, by mixing equal volumes of protein plus well solution. The RBD : ION_300 crystals grew in 16% PEG3350 and 0.3 M potassium citrate tribasic, whereas crystals of RBD : ION_360 crystals grew in 16% PEG3350 and 0.2 M ammonium citrate tribasic. For cryoprotection, crystals were generally transferred to a solution of mother liquor plus 22% ethylene glycol. X-ray diffraction data were collected by the autonomous European Synchrotron Radiation Facility (ESRF) beamline MASSIF-1 using automatic protocols for the location and optimal centering of crystals (13). Strategy calculations accounted for flux and crystal volume in the parameter prediction for complete datasets. Data from crystals of RBD : ION_300 and RBD : ION_360 were refined to 2.35 and 2.80 Å resolution, respectively. Data were processed using XDS (14) and AIMLESS from the CCP4 Suite (15). Cell parameters and data statistics are summarized in **Table S5**.

Both complex crystal structures were solved by molecular replacement using Phaser (16) utilizing the coordinates from the SARS-CoV-2 RBD domain (PDB: 7JMP) and using homology models for ION_300 and ION_360 antibodies generated using SWISSMODEL (17) to model the heavy and light chains. Atomic models were built using Coot (18) and refined with Refmac (19), Phenix (20) and PDB_REDO (21). The refinement statistics for both structures are summarized in **Table S5**. The coordinates for RBD : ION_300 and RBD : ION_360 have been deposited to the PDB and given the codes 7BNV and 7NP1, respectively.

## Results

### Isolation of Anti-SARS-CoV-2 Antibodies From Patient Derived Libraries Using Phage Display Technology

Peripheral blood samples of 18 patients admitted to hospital with acute COVID-19 pneumonia were collected under informed consent. Patient demographics and clinical information relevant to their admission were also collected (**Table S2**). The patients experienced an average of 11 days (range 4-20 days) of symptoms prior to the day on which the blood sample was taken.

Initial B cell receptor (BCR) sequence analysis of the V_H_ repertoire of these patients revealed a strong convergent sequence signature. In order to link sequence data to information on binding properties, V_H_ populations from these donors were incorporated into phage display libraries in the form of single chain variable fragments (scFv). By one approach we constructed hybrid libraries (of size 9 x 10^8^ clones) where the variable heavy (V_H_) genes from the patient IgG repertoire were combined with a pre-existing library of variable light (V_L_) genes derived from healthy donors (6). The second strategy focused on creating fully patient-derived libraries (of size 1.5 x 10^9^ clones) by assembling V_H_s and V_L_s on a donor-by-donor basis. Phage particles rescued from these libraries were used to carry out three rounds of panning on monomeric RBD and S1 ectodomains. The stringency of panning in each round was increased by reducing the antigen concentration to enrich for higher affinity binders.

Following three rounds of panning, the selected populations from both sets of libraries were sub-cloned *en masse* into an IgG expression vector while maintaining V_H_-V_L_ pairing from the selected scFvs. A total of 1,498 clones were picked and expressed as IgG1 in Expi293F cells and screened using an affinity capture assay which eliminates the effect of expression variation between clones (4, 7). Of the 1,498 clones expressed, 589 antibodies originated from the phage panning against S1 and were screened against both RBD and S1, while the remaining 909 antibody clones from RBD panning were screened only on RBD. This screen yielded a total of 719 binders (48%). Surprisingly, a vast majority (87.3%) of the binders originating from S1 selections were also directed at RBD epitopes, resulting in a mere 46 non-RBD S1 binders. The preferential enrichment of antibodies to RBD over other S1 epitopes indicates that RBD is the key immunogenic region within the S1 protein. Although the further characterization of the S1 binders led to identification of neutralizing antibodies (albeit weak), these antibodies will not be discussed in detail in this manuscript.

### Functional, Biochemical and Biophysical Characterization of Anti-SARS-CoV-2 Antibodies

**Figure 2:**
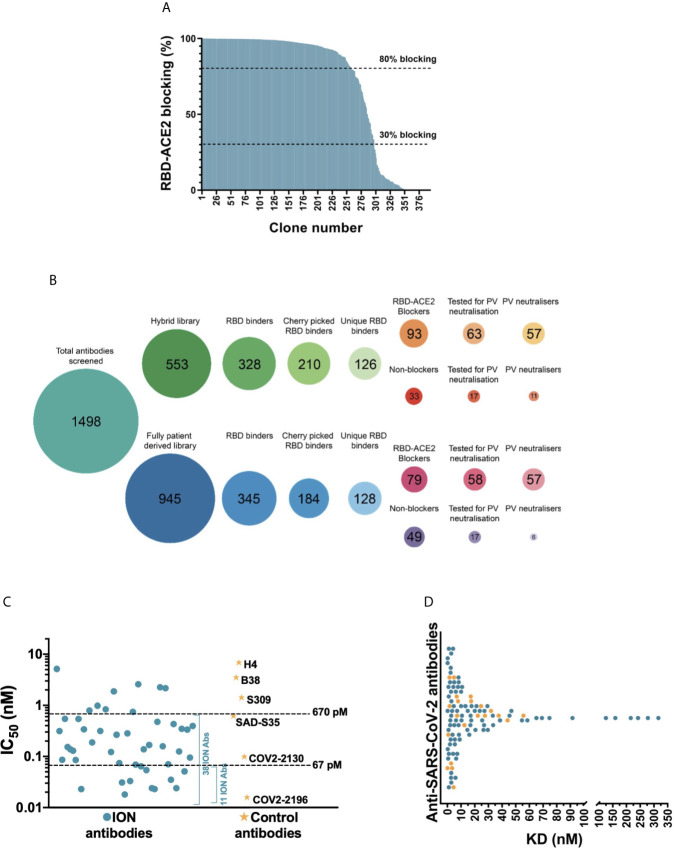
Biochemical and functional characterization of SARS-CoV-2 antibodies. **(A)** Screening of SARS-CoV-2 binders for RBD-ACE2 blocking activity. Antibodies were tested and ranked in a biochemical blocking assay for their ability to block the RBD-ACE2 interaction. **(B)** Antibody screening process presented as bubble plots. The number of antibodies tested from the hybrid and fully patient derived libraries at various stages of the study is highlighted inside each bubble. Areas of bubbles are scaled relative to each other based on the number of antibodies that represent each bubble. **(C)** Pseudovirus neutralizing activity of 52 SARS-CoV-2 antibodies and control antibodies. **(D)** Dot plot representing the 1:1 RBD binding affinities of SARS-CoV-2 antibodies measured using SPR. The antibodies that are part of the final panel of 21 are represented in orange dots. The break in the x-axis highlights the change in axis intervals.

It is reported that the primary mechanism of the most potent SARS-CoV-2 neutralizing antibodies is by blocking the RBD interaction with the ACE2 cell surface receptor (1, 22, 23). Therefore, we tested 394 RBD binders for their ability to block ACE2 in a biochemical assay (**Figure 2A**), while carrying out the Sanger sequencing of these clones in parallel. Of these, 254 antibodies had a unique V_H_ and V_L_ CDR3 sequence and 172 unique antibodies showed >30% blocking in the biochemical assay. From this category, 121 unique antibodies with highest binding and blocking activity were selected for viral neutralization assays. In addition, 34 clones with highest binding signals which failed to block the RBD-ACE2 interaction were progressed in the search for alternative mechanisms of action. This panel of 155 antibodies was subsequently purified from culture supernatants and screened in a lentiviral based SARS-CoV-2 pseudovirus assay at a concentration of 25 nM (3.5 µg/mL). 114 out of 121 (94.2%) ACE2 blocking antibodies showed >30% neutralization. Among this group, 97 (80.2%) antibodies showed neutralization activity exceeding 80%. Interestingly, a significant proportion of RBD-binding antibodies which failed to show ACE2 blocking in the biochemical assay (50%) showed neutralization of pseudovirus, albeit significantly weaker than the ACE2 blockers (with 9/34 antibodies showing >50% neutralization and only 1 antibody exceeding 80% neutralization). Selected antibodies from the two different library approaches (“hybrid” versus fully patient derived) performed equally well and produced a similar number of blockers and neutralizers (**Figure 2B**).

The half maximal inhibitory concentrations (IC_50_) of the top 52 antibodies were determined (**Figure 2C**). This experiment also included a number of recently published SARS-CoV-2 neutralizing antibodies including the potent antibody pair (COV2-2196 and COV2-2130) identified by the Vanderbilt University-AstraZeneca team (24). Of the 52 antibodies tested, 38 were categorized as strong neutralizers with IC_50_ values below 670 pM (100 ng/mL). Amongst the control antibodies, COV2-2196 and COV2-2130 demonstrated the best neutralization with IC_50_ titers of 16 pM and 99 pM respectively. 11 of the most potent antibodies neutralized the pseudovirus with IC_50_ ranging from 18-67 pM (2.7 ng/mL to 10 ng/mL) hence matching or exceeding the best antibodies reported (22, 24–28).

**Figure 3:**
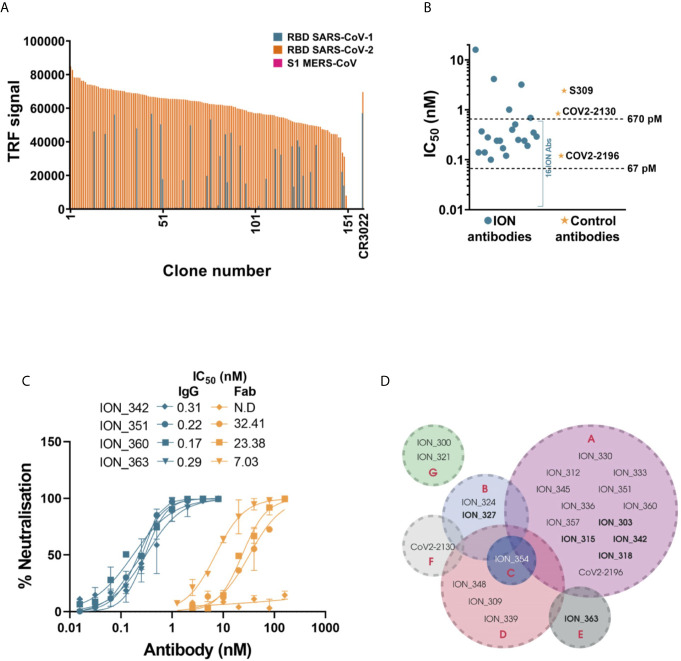
Detailed characterization of SARS-CoV-2 antibodies. **(A)** Evaluating the cross reactivity of 155 SARS-CoV-2 antibodies to SARS-CoV-1 RBD and MERS CoV S1. CR3022, a previously published binding SARS-CoV-1 and SARS-CoV-2 cross reactive antibody was used as control. **(B)** Authentic SARS-CoV-2 neutralizing activity of a panel of 21 SARS-CoV-2 antibodies and control antibodies. **(C)** Comparing authentic SARS-CoV-2 neutralizing activity of 4 antibodies, in Fab and IgG format. Error bars indicate standard deviation. **(D)** Epitope binning of SARS-CoV-2 antibody panel using Octet BioLayer interferometry. The bins are labeled A to G. ​Antibodies in bold text denote capture molecules that exhibited unidirectionality.

In parallel to the pseudovirus neutralization assay, the binding kinetics of 155 antibodies were determined by high-throughput surface plasmon resonance (SPR). The affinities of these antibodies ranged from 70 pM to 316 nM, with most antibodies clustered in the range of 1-30 nM (**Figure 2D** and **Table S3**). Comparison of the 52 clones tested for both affinity measurement and IC_50_ determination in pseudoviral neutralization assay, showed poor correlation between affinity and neutralization potency (**Figure S2A**). In addition, the cross-reactivity of these antibodies to SARS-CoV-1 and MERS-CoV were also evaluated using a binding assay based on time resolved fluorescence (TRF). Unsurprisingly, none of the antibodies recognized MERS-CoV S1 which has very low sequence homology with SARS-CoV-2 (~20% on RBD). Only the antibodies that bound to SARS-CoV-1 with binding signals within 5-fold of SARS-CoV-2 were considered as cross-reactives. 29/155 (18.7%) antibodies tested for cross-reactivity showed binding to SARS-CoV-1 RBD, which included 16 pseudovirus neutralizers (**Figure 3A**). Interestingly, a significantly higher proportion of non-ACE2 blocking antibodies showed cross reactivity to SARS-CoV-1 (55.9%) than the ACE2 blockers (8.3%).

Based on the potency of pseudovirus neutralization, a final panel of 21 antibodies were selected for further characterization (**Figure S1** and **Table S4**). Given the abundance of potent neutralizers, any antibodies with poor transient expression and sequence liabilities (deamidation motifs in CDRs, unpaired cysteines and glycosylation motifs in V regions) were excluded at this stage. This panel also included two non-blocking neutralizers, which despite showing only moderate neutralization in the pseudovirus assay were included due to their distinct mechanism of neutralization. Initially, these antibodies were evaluated for their ability to neutralize an authentic SARS-CoV-2 strain. A strong correlation was observed between the pseudovirus and real virus neutralization potencies (**Figure S2B**). 16/19 ACE2 blocking antibodies (**Figure 3B** and **Figure S3** and **Table S4**) neutralized the authentic virus with IC_50_ ranging from 100 pM (15 ng/mL) to ~670 pM (100 ng/mL). The control antibodies COV2-2196, COV2-2130 and S309 neutralized the virus with IC_50_s of 120 pM, 840 pM and 2.4 nM, respectively. The two non-blocking neutralizers (ION_321 and ION_300) showed IC_50_s of 4.15 nM and 16 nM, respectively. Finally, four potent antibodies were tested as Fabs and IgGs to determine the importance of valency in virus neutralization. The neutralization mediated by the IgGs were orders of magnitude more potent than that of equivalent Fabs (**Figure 3C**), suggesting the likely bivalent engagement of both IgG arms. The increased virus neutralization potency of IgG is likely to be a result of enhanced viral engagement due to avidity but could also include crosslinking spike proteins leading to aggregation of virions or steric exclusion (29).

Given the occurrence of RBD mutations among the circulating SARS-CoV-2 isolates, it is desirable to use a therapeutic approach that includes a cocktail of two or more antibodies with different contact residues to mitigating the risk of escape mutants. If these combinations are also directed against non-competing epitopes this may also induce synergetic neutralization effects (30, 31). Therefore, the final panel of 21 neutralizing antibodies were subjected to an epitope binning experiment using Octet Bio-Layer Interferometry to identify binding sites on RBD and their inter-relationships. Two control antibodies (COV2-2130 and COV2-2196) were also included in the analysis. Based on the pairing patterns, 7 different epitope bins were identified (**Figure 3D** and **Figure S4**). The majority of ACE2 blocking antibodies clustered into Bin A, which overlapped with four other bins. However, within this group, Bin B/Bin E combination and Bin C/Bin E combination were non-competitive. As expected, Bin G, containing the two non-ACE2 blocking neutralizers, showed no overlap with any of the other bins, hence creating five additional pairings involving an antibody from each bin. Based on this, 10 combinations covering all non-competitive bins (except for antibodies from Bin D and F) were tested to identify antibodies that can be paired together in an antibody cocktail. Of the 10 combinations tested, only two combinations showed moderate synergy while two combinations were additive, and six combinations were antagonistic (**Table 1**). Importantly, ION_300 (a non-ACE2 blocker) from Bin G paired well with antibodies from other bins and was part of the two combinations that showed moderate synergy.

**Table 1:** Effect of antibody combinations in the neutralization of authentic SARS-CoV-2.

Antibody 1	Antibody 2	Combination Index [CI]
ION_351	ION_300	1.7
ION_351	ION_354	5.4
ION_363	ION_354	1.8
ION_363	ION_300	**0.7**
ION_363	ION_324	1.1
ION_354	ION_300	1.3
ION_324	ION_300	1.8
ION_303	ION_300	1.6
ION_345	ION_300	1.2
ION_342	ION_300	**0.6**
ION_360	ION_300	1.1

Each antibody in the combinations listed was tested individually or at 1:1 ratio with the pairing antibody in the authentic SARS-CoV-2 neutralization assay. The Combination Index (CI) for each antibody pair was calculated with CompuSyn (28, 32). Based on the recommended cut-off values by CompuSyn, CI values <0.9 indicates synergy (in bold); 0.9<CI<1.1 indicate additive effect; CI>1.1 indicates antagonism. Antibody pair ION_351 and ION_354 from overlapping bins (A and C) was used as negative control for the assay.

Biophysical characterization of early-stage therapeutic candidates is important to identify any associated liabilities or risks that may hinder the progression of an antibody towards Chemistry, Manufacturing and Controls (CMC) (33). Although the functional binding properties set the threshold for progressing antibodies into preclinical and clinal development, good thermal, physical and chemical properties (collectively known as “developability” properties) are all required to ensure they can be produced at scale with minimal loss and are tractable as a potential human drug. Sub-optimal developability properties can cause poor *in vivo* efficacy, PK/PD and immunogenicity leading to expensive late-stage clinical failures or high costs of manufacturing (34, 35). Therefore, the final panel of neutralizing antibodies were subjected to a series of experiments to determine the developability profile (see **Supplementary Materials**). This includes a pH stress test (to mimic the virus inactivation step during manufacturing), thermal stress test, freeze-thaw test, fragmentation analysis using CE-SDS, purity and column interaction test using HPLC-SEC, propensity for self-aggregation using AC-SINS and determination of isoelectric points using capillary isoelectric focusing (cIEF). 13 out of 21 (60%) antibodies passed the cut-off values for each assay and were hence deemed developable (**Table 2**). This represents an attrition rate of 40% from the initial panel of 21 neutralizing antibodies tested, underlining the importance of developability testing as part of early antibody drug discovery.

**Table 2:** Summary of developability data for final panel of 21 antibodies.

Antibody ID	Freeze-thaw stress	pH stress	Thermal stress	Capillary isoelectric focusing (cIEF)		Non-reduced CE-SDS		HPLC-SEC	AC-SINS shift (nm)	Overall ranking
	Loss (%)	Loss after Protein A (%)	Tm (°C)	Main group (pI)		Glycosylated intact antibody (%)	Multiple species			
				Lower	Upper					
**ION_300**	0	4.3	63.2	8.46	8.63	97.6	No	S	7	PASS
**ION_303**	0.8	4.2	68.9	6.84	7.02	93.4	No	S	5	FAIL
**ION_309**	0.1	2.5	73.9	8.58	8.66	91.9	No	S	11	PASS
**ION_312**	0.8	9.3	70.6	8.31	8.41	90.1	No	S	4	PASS
**ION_315**	1.3	4.8	68.5	7.55	7.66	94.4	No	S	4	PASS
**ION_318**	0	1.9	71.3	8.77	8.83	87.2	No	D	29	FAIL
**ION_321**	0	3.6	64.1	8.45	8.60	68.6	Yes	S	7	FAIL
**ION_324**	0	1	64.7	7.72	7.93	67.6	Yes	S	4	FAIL
**ION_327**	0	5.4	67	7.45	7.58	60.4	Yes	D	7	FAIL
**ION_330**	0	2.4	62.8	7.72	7.82	96.5	No	S	6	PASS
**ION_333**	0.3	1.2	74.5	7.32	7.38	86.1	No	S	4	FAIL
**ION_336**	0.3	3.9	74.2	8.47	8.57	93.9	No	S	4	PASS
**ION_339**	0.9	6.2	67.9	7.63	7.83	95.1	No	S	17	PASS
**ION_342**	0.5	3.4	72.3	8.96	9.08	94.9	No	S	5	PASS
**ION_345**	1	2.6	68.6	8.37	8.49	91.4	No	S	4	PASS
**ION_348**	0.3	11.3	69	7.55	7.65	95.0	No	S	4	FAIL
**ION_351**	0	2.4	71.1	8.58	8.74	93.1	No	S	4	PASS
**ION_354**	0.3	3	77.1	8.93	9.08	94.0	No	S	5	PASS
**ION_357**	0.5	2.4	68.6	9.00	9.11	90.8	No	S	4	PASS
**ION_360**	0.4	4.7	65.1	8.85	9.01	90.2	No	S	7	PASS
**ION_363**	0	5.3	63.3	6.83	6.91	67.7	Yes	S	9	FAIL

Cut-offs were given for each test to decide what defines a “passing” antibody: <5% freeze-thaw loss, <10% loss after protein A, Tm >60℃, lower (<7.5) and upper pI species (>9.5), >90% intact glycosylated antibody and no presence of multiple species in CE-SDS, a standard (S) or delayed (D) HPLC-SEC profile, AC-SINS shift <20 nm. ​Antibodies that passed criteria set were given a green fill color; antibodies that failed the criteria set were given an orange color. A yellow fill color was given where antibodies were considered weaker but were not classified as failed. An overall developability pass or fail ranking was given based on all criteria.

### Crystal Structures of ACE-2 Blocking and Non-Blocking Antibodies in Complex With RBD

**Figure 4:**
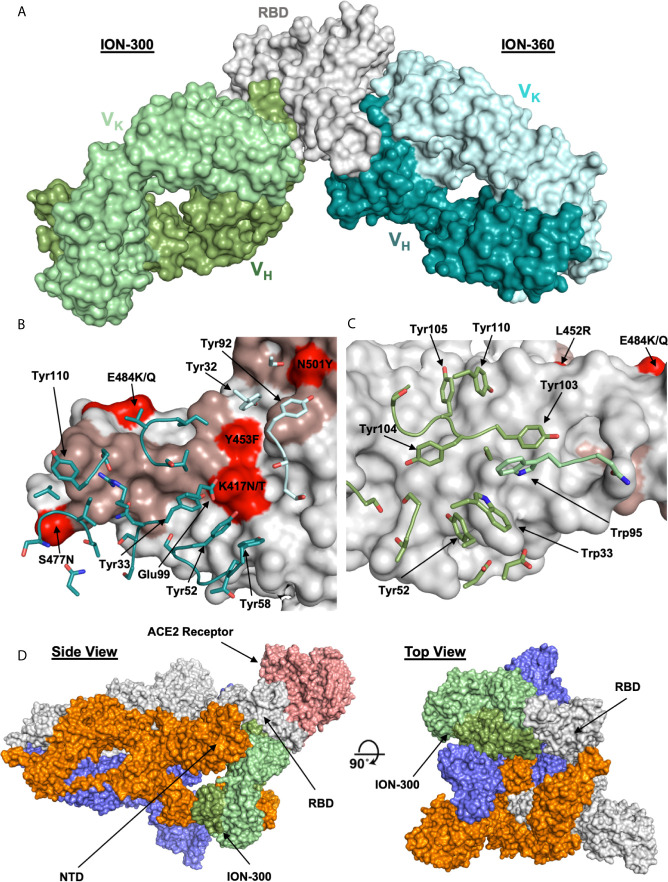
Structures of ION_300 and ION_360 antibodies bound to SARS-CoV-2 RBD. **(A)** Molecular surface representation of ION_300 (greens) and ION_360 (blues) overlayed by their bound RBD (grey). **(B)** Analysis of the RBD : ION_360 interface. CDR residues within 5 Å of the RBD are shown in sticks for the VH (blue) and VK (pale blue) chains. **(C)** Analysis of RBD:ION_300. CDR residues within 5 Å of the RBD are shown in sticks for the VH (green) and VK (pale green) chains. In both B and C RBD is represented as a surface (grey) with ACE2 binding residues highlighted (salmon). RBD mutations found within the three major SARS-CoV-2 variants (B.1.1.7, B.1.351 and B.1.1.28) are highlighted in red. **(D)** Overlay of the ION_300:RBD complex (greens) onto a published cryo-EM structure of the spike protein trimer (grey, orange and blue) bound to the ACE2 receptor (salmon) (PDB: 7a96), shown in molecular surface representation. Figures prepared using PyMol.

To understand and characterize the molecular basis of viral inhibition, we determined the crystal structure of the potent ACE2 blocking ION_360 bound to RBD and of the complementary non-receptor competitive ION_300 in complex with RBD. The structure of the ION_360-SARS-CoV-2 RBD complex was determined at 2.80 Å (**Figure 4A**). Consistent with its antiviral potency, ION_360 binds to the ACE2 receptor binding motif (RBM) on the SARS-CoV-2 RBD, which is a feature reported for a number of antiviral antibodies (27, 36). The RBD : ION_360 interface buries 851 Å^2^ of RBD protein surface from bulk solvent following complex formation. Of the 20 RBD residues within 5 Å of the ACE2 receptor that make up the binding site, 13 are buried upon ION_360 binding. In contrast, 859 Å^2^ of antibody surface is buried from bulk solvent, with 712 Å^2^ of the buried surface from the V_H_ domain and 147 Å^2^ from the V_K_ domain. With the exception of CDR-L2, at least one residue from all CDR loops is within 5 Å of the SARS-CoV-2 RBD, with 22 amino acids from the CDR loops losing at least 10 Å^2^ of solvent accessibility and making direct contacts with the protein. The interface features a mixture of polar and hydrophobic contacts, including the involvement of six aromatic side chains in the CDR loops, from both the V_H _(Tyr33, Tyr52, Tyr58, Tyr110) and V_K_ (Tyr32 and Tyr92) domains, as well as a network of 13 hydrogen and salt bonds, accounting for a measured K_D_ of 1.5 nM.

Several pre-clinical and clinical anti-SARS-CoV-2 antibodies targeting RBM were shown to be affected by K417N/T, E484K and N501Y RBD mutations found in the recent virulent strains (B.1.1.7, B.1.351, and B.1.1.28) identified in the UK, South Africa and Brazil respectively (37). The analysis of the RBD: ION_360 interface suggests these mutations are unlikely to affect neutralization potency of ION_360 (**Figure 4B**). E484K and N501Y mutations are on the periphery of the interface and in excess of 6 Å distance from the ION_360 antibody paratope, thus minimizing their involvement in the interaction. Whilst Lys417 is making a potential salt bridge to Glu99 from CDR-H3, the subtle reduction in residue volume of the K417N/T mutation, leading to the loss of a single salt bridge, is not expected to impact on the interaction of ION_360 with the RBD from these strains.

To determine the mechanism of viral neutralization of the non-ACE2 blocking antibody ION_300, the structure of the ION_300-SARS-CoV-2 RBD complex was solved by protein crystallography to 2.35 Å. Our structure revealed that the ION_300 interface with the RBD is predominantly through the V_H_ domain, burying 647 Å^2^ of antibody surface, compared to only 27 Å^2^ for the V_K_ domain. All three CDR loops from the V_H_ domain contribute at least three residues to the interface, whereas only two residues from the V_K_ domain are involved, with a total of 19 residues across both domains losing at least 10 Å^2^ of solvent accessibility following binding. The contact surface involves seven aromatic residues from ION_300 CDR loops, three of which are central to CDRH3 interactions (Tyr103, Tyr104, Tyr105), as well as seven hydrogen bonds and two salt bridges. An illustration of the RBD : ION_300 interface is shown in **Figures 4A, C, D**. The structure of the complex reveals that ION_300 binds away from the receptor binding motif recognized by ACE2 on the RBD and against the exposed ß-pleated sheet, particularly impacting on residues K462-S469 immediately following the ß1’ strand.

It is important to note that the epitope identified for ION_300 is distinct from all current published anti-SARS-CoV-2 antibodies (**Figure S5**). There is a very limited overlap with the epitope of Fab 52 [PDB: 7k9z (38)], which contacts the backside of the RBM, whereas ION_300 shows no contact with the RBM. Furthermore, the mutations associated with the recent virulent strains B.1.1.7, B.1.351, and B.1.1.28 (K417N/T, E484K and N501Y) and other prevalent RBD mutations reported previously (V367F, N439K, Y453F, S477N, V483A (39), are not found within the epitope of ION_300. The closest mutation (E484K) is at least 15Å away from the paratope of ION_300. Therefore, these mutations are considered highly unlikely to affect the antiviral potency of this antibody. Nevertheless, these structure-based predictions require validation in neutralization assays using viral variants.

Structural alignment of the RBD from the ION_300 complex with the RBD from the ACE2 complex (PDB:6m0j) reveals very limited conformational differences, with an overall rmsd of 0.76 Å when aligning all Ca atoms, strongly suggesting that the antiviral mechanism of action of ION_300 is not allosteric. Furthermore, in reported cryo-EM structures of the SARS-CoV-2 spike protein, the ION_300 epitope on the RBD is buried behind the NTD of an adjacent S1 polypeptide chain when the RBD is in the closed conformation. The ION_300 binding site only becomes accessible when the RBD is in the open conformation (**Figure 4C**), indicating that binding of this antibody may result in a spike protein which is locked in an RBD open type conformation. This perhaps suggests that ION_300 may utilize a similar ratcheting mechanism to that proposed by David Veesler’s Group (32, 40, 41), in which the open/closed equilibrium of the spike protein, with regard to the RBD, is pushed to a fully open conformation following antibody binding. Such changes may cause a premature adoption of a post-fusion spike conformation, ultimately resulting in the inhibition of virus entry into host cells. Alternatively, steric hindrance or aggregation of virions *via* bivalent crosslinking of spike trimer could also contribute to viral neutralization function of ION_300 (29).

### RBD-Binding Antibodies Arise From Recent B Cell Activation and Correlate With Patient Status

The BCR repertoire sequence data from these patients has been previously published (5), yielding 3,485,995 unique V_H_ sequences across all patients. To investigate the B cell responses of the patients in more detail, the sequence data from the 254 unique RBD-binding antibodies isolated by phage display were integrated with the total BCR repertoire data (**Figure 1**). Co-clustering of the two datasets was performed using a previously described threshold (see *Methods*) to group together sequences that are sufficiently similar to be considered part of the same B cell clonal expansion, and likely targeting the same epitope. This clustering yielded 838,887 clusters across all of the patients; these clusters were then labeled according to whether they contained any of the RBD-binding antibodies identified from the phage display process. Of the 254 unique RBD-binding antibodies, 201 of these co-clustered with the total BCR sequence data. Several of these 201 antibodies mapped to the same cluster, resulting in 89 different clusters that could then be annotated as RBD-binding. 108 of the 201 RBD-binding antibodies also showed >30% neutralizing activity in the pseudoviral neutralization assay (at 25 nM), enabling 49/89 of the RBD-binding clusters to also be annotated as containing neutralizing antibodies.

**Figure 5:**
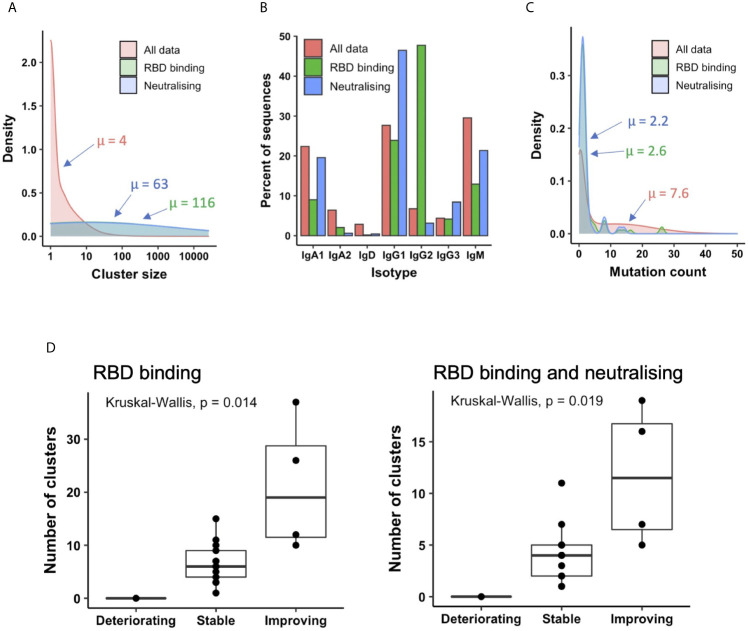
Relating antibodies discovered by phage display back to patient B cell repertoire data. **(A)** Sequences clustered into related groups. Clusters then annotated based on whether they contained RBD-binding or its subset of neutralizing antibody sequences. Density plot shows distribution of clusters of different sizes in the combined dataset from all 18 patients. **(B)** The isotype subclass distribution of sequences belonging to the different groups of clusters. **(C)** Mean mutation of all sequences within each cluster was calculated. Density plot shows the distribution of clusters with different numbers of mutations. **(D)** The box plot shows the number of clusters annotated as RBD binding (left), or RBD binding and neutralizing (right) in each patient, stratified according to disease status.

The mean size of all clusters across patients was four sequences, but the clusters annotated as containing RBD-binding or neutralizing antibodies contained on average 116 and 63 sequences, respectively, indicating that these B cells are undergoing clonal expansion (**Figure 5A**). Investigating the isotype subclass distributions of the sequences within these clusters showed different distributions for total clusters, clusters annotated as RBD-binding, and clusters annotated as RBD binding and neutralizing (a subset of RBD binders that neutralize the virus, **Figure 5B**). 71% of RBD-binding clusters, and 80% of neutralizing clusters contained IgM sequences, indicating recent activation of these B cells. Furthermore, calculating the mean mutation from germline of the sequences within the clusters showed that the RBD-binding and neutralizing clusters had fewer mutations than total clusters (RBD-binding: 2.6, Neutralizing: 2.2, Total: 7.6), giving further evidence that the RBD-binding and neutralizing clusters have arisen from recently activated B cells (**Figure 5C**).

Looking at total clusters, there is a bimodal distribution of mutation counts, which becomes more pronounced when considering the clusters that do not contain IgM sequences (**Figure S6A**), and those with evidence for clonal expansion (**Figure S6B**). We interpret the first mode of the distribution to represent naïve or recently activated B cells, and the second mode to represent memory B cells. By cutting this bimodal distribution we can therefore classify whether the RBD-binding clusters had arisen from a naïve response, or memory recall. An arbitrary threshold of a minimum of 5 mutations was chosen to classify a cluster as having arisen from memory recall based on manual inspection of histogram, and selection of a value that best separates the two modes. In total, 88% of the RBD-binding clusters, and 90% of the neutralizing clusters were classified as having arisen from recent activation. We can thus conclude that the response to COVID-19 is largely driven by naïve B cell activation, and there is very little re-activation of circulating memory cells.

Next, the number of clusters annotated as either RBD-binding or neutralizing was calculated independently for each patient. There were no RBD-binding clusters identified in the one deteriorating patient (**Figure 5D**), but at least one identified in every stable and improving patient. There were significant differences in the number of both RBD-binding and neutralizing clusters identified between each of the three patient groups. There were more RBD-binding and neutralizing clusters identified in the improving compared to the stable, and the stable compared to the deteriorating groups. Improving patients therefore provide the best source for therapeutic antibody discovery; the deteriorating patients may have yet to mount an effective immune response.

### The Antibody Response to COVID-19 Is Highly Convergent

We and others have previously observed a stereotypic BCR response to COVID-19 infection (5). We replicate the findings here, showing that the clusters annotated as RBD-binding use a restricted set of V gene segments (**Figure S7**). Clusters utilizing VH3-30, VH3-53, VH3-66, VH3-9 and VH5-51 dominated the response, with lower levels of VH1-18, VH1-2, VH1-49, VH1-69, VH3-11, VH3-13, VH3-15, VH3-20, VH3-23, VH3-48, VH3-64 and VH3-7 also seen. Heavy and light chain V gene utilization among the RBD binders identified from phage display screening is shown in **Figure S8**.

We have now extended these observations by investigating the specific convergence between individuals of the clusters annotated as RBD-binding or neutralizing. Of the 89 clusters annotated as RBD-binding, 26% (23/89) of the RBD binders and 33% (16/49) of the neutralizing clusters were convergent across at least two individuals. This is in stark contrast to the convergence seen across the entire dataset which is 2.5% of total clusters. Furthermore, one of the RBD-binding and neutralizing clusters was shared between 14 of the 18 different individuals in the study (Cluster ID 3, **Table 3A**, and **Table S6**). Interestingly, the moderately neutralizing non-ACE2 blocking antibodies were also abundant and convergent with one clonally expanded cluster (size = 128) recurring among seven individuals.

**Table 3A:** Convergent clusters identified in the current study which also had sequences map to them from the CoV-AbDab.

Convergent cluster ID	Representative CDRH3	V gene	J gene	Cluster size	Mean mutation	Convergence	Number of CoV-AbDab hits
1	AAPDCSSTSCYDAFDI	VH1-58	J3	1680	1.7	9	8 mAbs
2	ARDLVAYGMDV	VH3-66	J6	164	2	9	10 mAbs
3	ARDLMVYGMDV	VH3-53	J6	1202	2.1	14	14 mAbs
4	ARDAMSYGMDV	VH3-53	J6	71	0.9	4	2 mAbs
5	ASSLWLRGSFDY	VH3-7	J4	45	1.1	3	1 mAbs
6	AGGPNLNNWFDP	VH5-51	J5	72	1.6	3	1 mAbs
7	ARDLDVRGGMDV	VH3-66	J6	43	1.9	3	2 mAbs

**Table 3A** shows the properties of convergent antibody clusters identified from the current study, including the size of the cluster (i.e., unique antibodies found in each cluster, an indicator of clonal expansion), number of different patients each cluster was present in (convergence) and number of antibodies found in the CoV-AbDab database with the same cluster identity.

In addition to investigating the convergence between individuals in the current study, we also investigated the convergence with SARS-CoV-1/2 binding antibodies identified in other studies. We extracted all V_H_ sequences from the CoV-AbDab (3) annotated as having a human-derived V gene segment, and having V and J gene segment annotations, and CDRH3 sequence determined. This yielded 1,879 unique sequences. These sequences were integrated into the clustering analysis of phage-derived RBD-binding and neutralizing antibodies, and the total BCR repertoire data. In total, 244 of the clusters in the total BCR repertoire contained antibodies that had been extracted from the CoV-AbDab, showing that convergence is seen between studies. Furthermore, there were seven clusters which contained both antibodies from the CoV-AbDab and antibodies from the current study (**Table 3A**). All of these clusters were classified as having arisen from recent activation based on having <5 mean mutations, and the presence of IgM sequences. These clusters were all convergent between at least three individuals in the current study. Two of these clusters (Cluster ID1 and Cluster ID2) were also convergent between nine individuals in this study, as well as three separate studies from the CoV-AbDab. Both of these clusters were in the panel of the 21 most potent antibodies discovered in the current study. For example, antibodies ION_312 and ION_1000, were highly similar to a clonally expanded sequence cluster (containing 1,680 unique sequences, Cluster ID1, **Table 3A**) from nine different patients in this study and antibodies from four separate studies (**Table 3B**), indicating its importance in mediating protection.

**Table 3B:** Convergence of antibody sequences across separate studies.

Antibody ID	CDRH3	V gene	J gene	Reference
ION_312	AAPDCSSTSCYDAFDI	VH1-58	J3	Current study
ION_1000	- - -H- - - - - -N- - - - -	VH1-58	J3	Current study
C005	- - -H- -GG- -L- - - - -	VH1-58	J3	Robbiani et al. (42)
COV2-2381	- - -Y- -R- - -H- - - - -	VH1-58	J3	Zost et al. (24)
CV07-287	- - -Y- - - -N- - - - - - -	VH1-58	J3	Kreye et al. (43)
HbnC3t1p_C6	- - -Y- - - -R- - - - - - -	VH1-58	J3	Kreer et al. (44)
ION_336	ARDLVAYGMDV	VH3-66	J6	Current study
BD-498	- - - - -V- - - - -	VH3-66	J6	Cao et al. (23)
C140	- - - -YY- - - - -	VH3-66	J6	Robbiani et al. (42)
COV2-2080	- - - - -T- -L- -	VH3-66	J6	Zost et al. (25)

Heavy CDR3 sequence similarity (>80%), V gene, J gene usage shown between antibodies identified within the current study to those in separate published studies.

## Discussion

We have previously described (5) B cell receptor repertoire analysis of the V_H_ populations from 18 SARS-CoV-2 positive donors at an acute stage of disease. In the present study we have constructed libraries from the antibody repertoire of these patients and used phage display to identify a sub-set of antibody genes which encode functional binders and potent neutralizers of SARS-CoV-2. Unlike most direct B cell screening methods, phage display technology allows processing of millions of antibody genes from large numbers of patient donors. It also allows enrichment of functional clones that may be rare or have not yet undergone clonal expansion within the initial antibody response. This deep mining approach yielded neutralizers to diverse epitopes within the RBD. This includes a group of highly potent antibodies which compete with ACE2 for RBD binding, with neutralization IC_50s_ matching the best antibodies reported with similar mechanism of action (22, 24, 25, 28, 45).

Another group included antibodies with moderate potencies which neutralized the virus through a distinct mechanism of action (independent of inhibiting the RBD-ACE2 interaction) including an antibody to a unique epitope (ION_300). Despite their relatively weaker potency, non-ACE2 blocking neutralizers were abundant within the clonally expanded patient antibody response with strong convergence scores, indicating that such antibodies also play a key role in providing protection. The crystal structure of ION_300 from this group revealed that it binds to a unique epitope at the opposite side of the RBM. Identification of contact residues within the epitope predicts that this antibody is likely to retain binding and neutralization potency to the RBD mutations that enhance the infectivity of the widely circulating “Kent”, “South African” and “Brazil” variants (B.1.1.7, B.1.351 and B.1.1.28 respectively). In addition, other reported (albeit less prevalent) RBD mutations V367F, N439K, Y453F, S477N, V483A, are also not found within the epitope of ION_300. Furthermore, ION_300 paired well with potent antibodies from ACE2-competing epitope bins including ION_360 which is also unlikely to be affected by the three major variants, providing a compelling case for further optimization and inclusion in an antibody cocktail for *in vivo* evaluation. As ION_300 V_K_ domain makes limited contact with the RBD, there is scope to further enhance potency using commonly applied antibody engineering techniques such as light chain shuffling or targeted mutagenesis of CDRs to achieve greater epitope coverage. However, it remains an open question whether increased selection pressure arising from widespread use of antibodies such as ION_300 will also result in emergence of escape mutants. It is worth noting that a significantly higher proportion of non-ACE2 blocking antibodies (including ION_300) showed cross-reactivity to SARS-CoV-1 (55.9%) in comparison to the ACE2 blockers (8.3%), indicating the relative sequence conservation within non-RBM epitopes. Taken together, non-ACE2 blocking RBD neutralizers such as ION_300 may have a greater potential for being developed as broadly neutralizing antibodies to mutant SARS-CoV-2 strains than the antibodies targeting RBM epitopes.

Sequence analysis of the selected functional binders from this study shows that many of the potent antibodies are identical to, or within a few amino acids of, germline encoded V and J sequences. This is in line with previous reports from Kreer et al, who describe isolation of neutralizing IgG antibodies with a spectrum of variable domains with low levels of somatic mutation derived from memory B cells (44). Projection of the sequences of the confirmed RBD binders and neutralizers identified in this study onto a dataset derived from the same patient group including V_H_s from both IgG and IgM populations, confirms that a number of these potent neutralizers (originally selected from the IgG pool) are in fact represented in the IgM repertoire. Thus, the occurrence at an early stage of infection of relatively unmutated V_H_ genes within both IgM and IgG populations is indicative of recently class-switched B cells that have yet to go undergo somatic hypermutation.

By combining the identification of highly potent neutralizing antibodies with deep sequencing we highlight convergence within the antibody response among different patients within this study group and beyond. The occurrence of a convergent antibody response among COVID-19 patients is described to a lesser extent by other studies. For example, Robbiani et al. purified IgG-expressing, RBD-binding memory B cells from a limited number of convalescent COVID-19 patients (n=6) at an average of 39 days post-symptom onset (42). Analysis of the antibody genes revealed the presence of closely related antibodies in different individuals at this later time point in the memory pool. Here, we show evidence of recurring antibody genes within the total antibody pool (including the IgM repertoire) of 18 patients and also among the published anti-SARS-CoV-2 antibody sequences from multiple independent studies, including one of the most convergent sequences from Robbiani et al. (**Table 3B**).

Convergent antibody responses have been described in the response to other infectious diseases (46–48), but to our knowledge, the level of convergence seen in the COVID-19 response has not been reported in other disease settings. The occurrence of potent neutralizing antibodies within the germline encoded naïve repertoire may be part of the explanation. The high convergence of specific BCR sequences in COVID-19 that have protective properties suggests that developing these into antibody therapeutics could be highly effective. Monitoring for the development of these sequences may also be used as a generic method for assessing efficacy of novel vaccine strategies.

The majority of characterized antibodies from these patient derived libraries had affinities in the range of 1-30 nM, which is typical of antibodies isolated from IgM-derived naïve phage display repertoires (49–51) and did not show any strong correlation with neutralization potency. The ability of a naïve-like antibody response with moderate binding affinities to impart highly potent viral neutralization could be rationalized by the avid binding of bivalent IgGs to the trimeric spike protein (the main target of neutralizing antibodies reported here or elsewhere). This could also explain the comparable performance of hybrid libraries (where the patient derived V_H_s were paired with naive/unmutated V_L_s from healthy donors) and fully patient derived libraries (where both V regions were isolated from COVID-19 patients). The importance of valency in neutralization is demonstrated by the comparison of neutralization IC_50_s of IgGs and Fabs (**Figure 3C**).

While the devastating effect of SARS-CoV-2 infection on the population is clear to see, this study clearly demonstrates the presence of potent neutralizing antibodies within the naïve repertoire. This readiness may be explained by the combination of high diversity derived from the antibody germline locus together with a multivalent presentation of antibodies effecting potent neutralization of polyvalent targets. Furthermore, the early antibody response in COVID-19 is highly convergent and can be mined for therapeutic candidates with broad neutralization potential against widely circulating SARS-CoV-2 strains.

## Data Availability Statement

Materials and sequences generated in this study will be made available on request (email:jmc@iontas.co.uk), but we will require a completed Materials Transfer Agreement signed with IONTAS Ltd. Structures generated in this study have been deposited to the Protein Data Bank with codes 7BNV and 7NP1.

## Ethics Statement

The studies involving human participants were reviewed and approved by NHS HRA RES Ethics. The patients/participants provided their written informed consent to participate in this study.

## Author Contributions 

AK-V, JM, JO, and JG conceived the study. PP and W-YL recruited the patients and executed clinical protocols. AK-V, KS, JG, LM, PV, SS, MC, SH, DM, JC, GM and OA designed experiments. GB, PV, LM, LL, EM, KP, KC, SE-C, DP, LC, CT, and ID carried out the antibody selection and screening. RL, PV, EM, DTa, DTo, SM, and RB carried out expression, purification and biophysical characterization. GM and EB performed neutralization assays. GH and MB performed the structural studies. AK-V, GB, JG, JM, LL, LM, GH and KS wrote the manuscript. GB and JG contributed equally to the work. All authors contributed to the article and approved the submitted version.

## Conflict of Interest

GB, PV, LM, LL, EWM, ID, KP, CT, RL, KC, SE-C, DP, SBS, JMcC and AK-V were employed by IONTAS Ltd. JDG and JO were employed by Alchemab Therapeutics Ltd. OA, LC and JIC were employed by Abcam. This work has been described in provisional patent applications.

The remaining authors declare that the research was conducted in the absence of any commercial or financial relationships that could be construed as a potential conflict of interest.
