# Supplementary material for: Cross-Reactive SARS-CoV-2 Neutralizing Antibodies From Deep Mining of Early Patient Responses
Source: Front Immunol. 2021 Jun 15;12:678570. doi: 10.3389/fimmu.2021.678570 (PMC8239432; doi:10.3389/fimmu.2021.678570)
Supplement: Supplementary file 1 [file DataSheet_1.pdf]

Supplementary Material

Supplementary Methods

Developability assessment of anti-SARS-CoV-2 antibodies

pH stress test

Affinity-captured samples were eluted from a 0.4 mL HiTrap Fibro PrismA unit using 0.1 M sodium citrate (pH 3.5), held for at least 30 minutes (virus inactivation) and neutralized using 1 M Tris-HCl (pH 9) in a 4 : 1 (sample: Tris-HCl) ratio. Analytical samples (10 µL) were taken and injected onto a Superdex 200 Increase 5/150 (Cytiva) using an Agilent 1260 Infinity HPLC system. Detection of aggregates was performed using a multi-angle light scattering (MALS) detector (DAWN HELEOS; Wyatt technology) and refractometer (Optilab TRex; Wyatt technology) coupled in-series to the UV detector. Peak analysis was performed using the software, Astra (Wyatt technology).

Determination of melting temperature

Melting temperatures (Tm) were determined by monitoring changes in intrinsic fluorescence (300 – 430 nm) versus temperature using the Uncle (Unchained labs). 9 µL samples (1 mg/mL) in PBS were thermally ramped from 25°C to 95°C (0.5 °C/min) and measured in triplicate. Analysis of the fluorescence signal was performed using the barycentric mean (BCM) and Tm values calculated by taking the first derivative, as determined by the associated software package.

Freeze-thaw stress test

Samples (1 mg/mL) in PBS were subject to five freeze-thaw cycles by placing samples into a minus 80 °C freezer (30 mins) and thawing at room temperature (15 mins). Analytical samples (10 µL) were taken before and afterwards and injected onto a Superdex 200 Increase 5/150 (Cytiva) using an Agilent 1260 Infinity HPLC system. Detection of aggregates was performed using a multi-angle light scattering (MALS) detector (DAWN HELEOS; Wyatt technology) and refractometer (Optilab TRex; Wyatt technology) coupled in-series to the UV detector. Peak analysis was performed using the software, Astra (Wyatt technology).

Capillary Isoelectric Focussing (cIEF)

All samples (8 µg) were desalted (< 50 mM NaCl) and added to a cIEF master mix (including pharmalyte and 1.5-3 M urea) in accordance with manufacturer instructions. The final sample was loaded onto a PA800 plus pharmaceutical analysis system with fitted capillary (neutral-coated) and required running reagents (Beckman Coulter). Analysis was performed using associated software.

CE-SDS

Samples (20 µg) were desalted (< 50 mM NaCl) and either alkylated (non-reduced) or reduced (with 2-mercaptoethanol) prior to heating samples for 10 mins at 70 °C, in accordance with manufacturer instructions. The final sample was loaded onto a PA800 plus pharmaceutical analysis system with fitted capillary (bare-fused silica) and required running reagents (Beckman Coulter). Analysis was performed using associated software.

HPLC-SEC and AC-SINS

Purified antibodies were run on HPLC-SEC (Agilent 1100) using a Superdex 200 Increase column, with 1X PBS running buffer and a flow rate of 0.25 mL/minute to assess the column retention time. The AC-SINS protocol used in the assay is previously described [51]. Antibodies were tested at a concentration of 10 µg/mL. Antibodies that showed >20 nm shift were classified as ‘poor’.

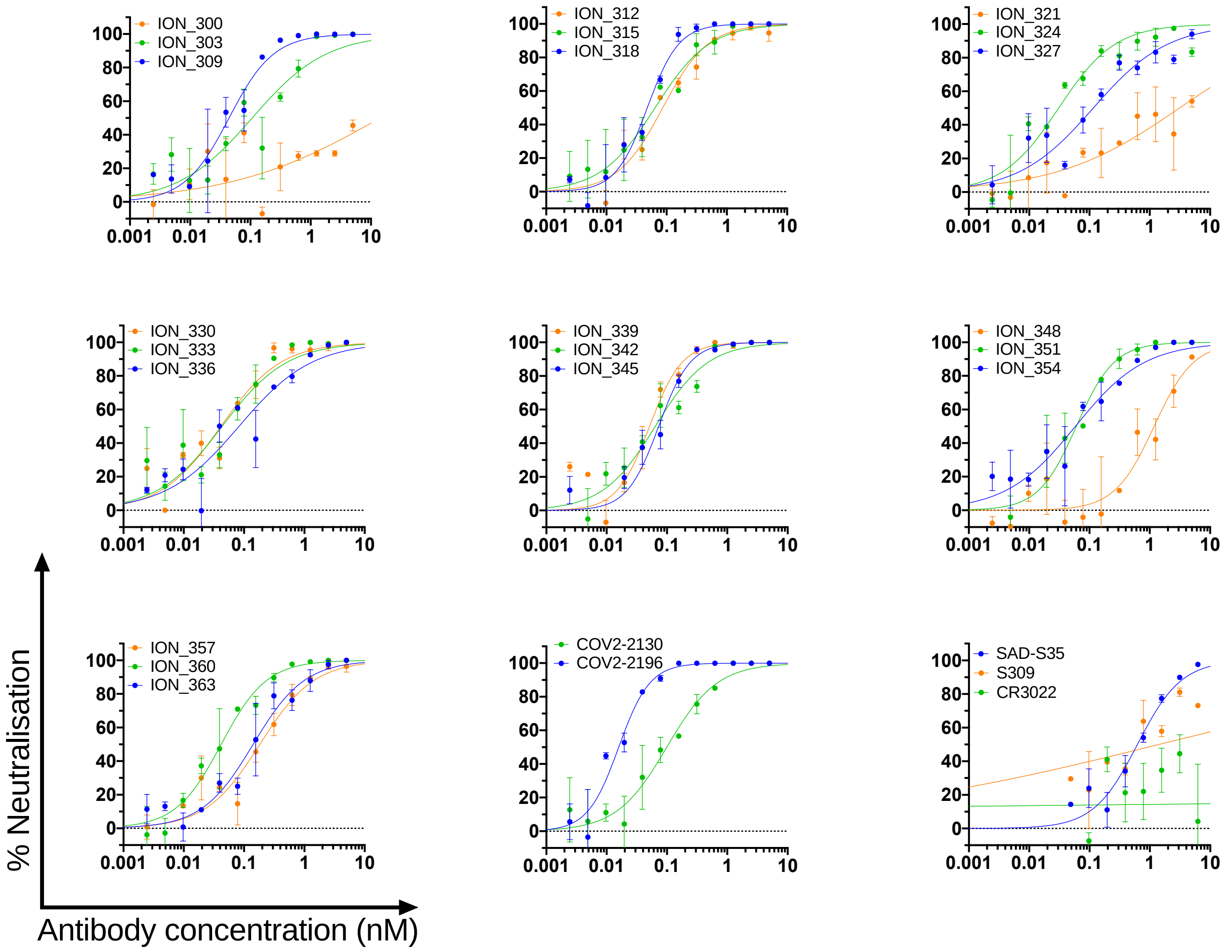

Figure S1. Dose-response curves demonstrating pseudovirus neutralization. The top panel of 21 antibodies were tested for pseudovirus neutralization. Control antibodies COV2-2130, COV2-2196, SAD-S35, S309 and CR3022 were included.

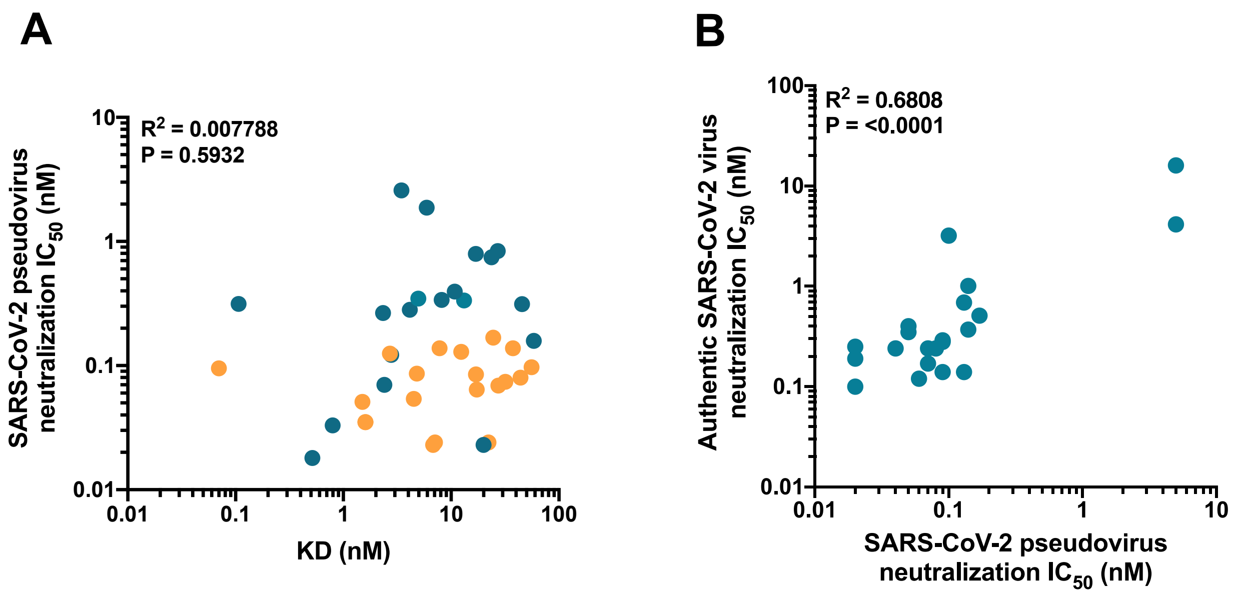

Figure S2. Correlation of pseudovirus neutralization with authentic virus neutralization and affinity. A) Correlation between pseudovirus neutralization and affinity. IC_{50} values plotted (nM) and KD (nM) for a panel of 39 antibodies, 19 of which are in the panel of the top 21 antibodies (shown in orange). B) Correlation between pseudovirus neutralization and authentic virus neutralization. IC_{50} values plotted (nM) for each neutralization assay for the final panel of 21 antibodies. R^2 and P values were determined via simple linear regression using GraphPad version 8.

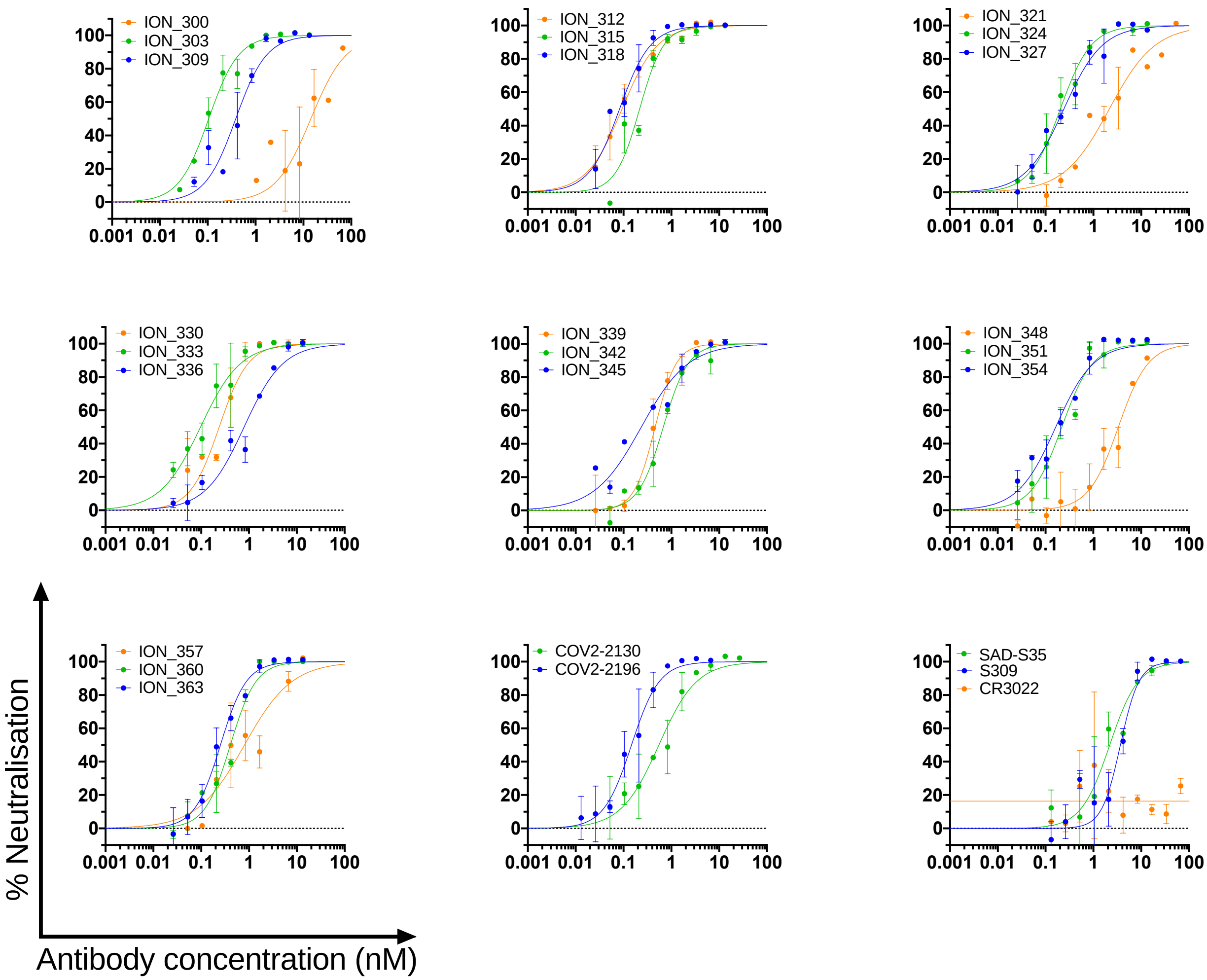

Figure S3. Dose-response curves demonstrating authentic virus neutralization. The top panel of 21 antibodies were tested for neutralization of the Australian isolate VIC01/2020 virus strain. Control antibodies COV2-2130, COV2-2196, SAD-S35, S309 and CR3022 were also included.

A

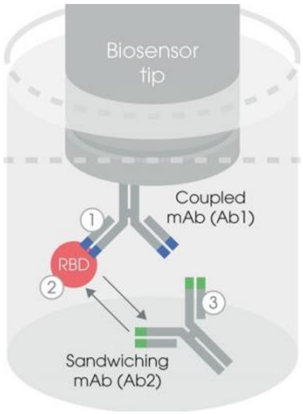

B

Antibody 2 (columns) / Antibody 1 (rows)

Clone	ION_309	ION_339	ION_348	CoV2-2196	ION_312	ION_330	ION_333	ION_336	ION_345	ION_351	ION_357	ION_360	**ION 303**	**ION 315**	**ION 342**	**ION 318**	ION_354	ION_324	**ION 327**	**ION 363**	CoV2-2130	ION_300	ION_321
ION_309	0.00	0.01	-0.03	-0.01	-0.01	0.02	0.00	0.00	-0.01	0.01	0.00	0.01	-0.01	-0.01	0.01	0.06	0.01	-0.01	-0.03	0.05	-0.02	0.31	0.29
ION_339	-0.03	0.00	-0.05	-0.03	-0.05	-0.01	-0.01	-0.02	-0.01	-0.01	-0.01	-0.02	-0.04	-0.05	-0.01	0.10	-0.01	-0.03	-0.05	0.02	-0.04	0.38	0.37
ION_348	0.00	0.00	-0.04	-0.01	-0.01	0.01	0.01	0.00	-0.01	0.01	-0.01	0.02	0.00	-0.01	0.02	0.08	0.01	-0.01	-0.02	0.08	-0.01	0.13	0.11
CoV2-2196	-0.02	-0.01	-0.05	-0.03	-0.03	-0.01	-0.02	-0.01	-0.02	0.00	-0.02	-0.01	-0.02	-0.03	0.00	0.04	0.00	-0.03	-0.03	0.02	0.17	0.24	0.23
ION_312	-0.02	-0.02	-0.04	-0.03	-0.03	0.00	-0.02	-0.01	-0.03	0.00	0.00	-0.01	-0.01	-0.03	0.00	0.04	-0.02	-0.03	-0.04	0.02	0.10	0.18	0.19
ION_330	-0.02	-0.01	-0.03	-0.02	-0.04	0.00	-0.01	-0.02	-0.02	-0.01	0.00	-0.01	-0.04	-0.04	0.00	0.04	0.02	-0.02	-0.03	0.02	0.19	0.29	0.27
ION_333	-0.03	-0.02	-0.05	-0.04	-0.04	-0.01	-0.02	-0.03	-0.03	-0.02	-0.01	-0.03	-0.03	-0.06	-0.01	0.08	0.00	-0.03	-0.05	0.00	0.18	0.33	0.37
ION_336	-0.03	-0.01	-0.05	-0.03	-0.04	0.00	-0.01	-0.02	-0.02	-0.01	-0.01	-0.03	-0.03	-0.05	-0.01	0.09	0.00	-0.02	-0.05	0.00	0.23	0.40	0.41
ION_345	-0.03	-0.02	-0.06	-0.04	-0.04	0.00	-0.01	-0.03	-0.03	-0.02	-0.01	-0.03	-0.03	-0.03	-0.02	0.03	-0.02	-0.03	-0.06	-0.02	0.11	0.22	0.22
ION_351	-0.04	-0.03	-0.06	-0.04	-0.05	-0.01	-0.01	0.05	-0.05	-0.02	-0.02	-0.04	-0.04	-0.05	-0.02	0.02	-0.01	-0.04	-0.05	-0.01	0.30	0.52	0.56
ION_357	-0.03	-0.02	-0.06	-0.03	-0.04	0.00	-0.01	0.01	-0.03	-0.02	-0.01	-0.03	-0.03	-0.04	0.01	0.03	0.00	-0.03	-0.04	0.00	0.37	0.50	0.55
ION_360	-0.04	-0.03	-0.06	-0.04	-0.03	-0.01	-0.01	0.02	-0.03	-0.02	-0.02	-0.04	-0.03	-0.04	-0.02	0.01	-0.02	-0.04	-0.06	-0.02	0.21	0.35	0.34
**ION 303**	-0.01	-0.01	-0.03	0.00	-0.01	0.01	0.00	-0.01	-0.02	0.01	0.00	0.01	0.00	-0.02	0.01	0.05	0.01	-0.02	-0.02	0.03	0.00	0.18	0.19
**ION 315**	-0.02	-0.02	-0.06	-0.04	-0.03	-0.01	0.00	0.01	-0.03	-0.01	-0.01	-0.02	-0.02	-0.03	-0.01	0.03	-0.02	-0.04	-0.05	0.00	0.07	0.16	0.18
**ION 342**	-0.03	-0.01	-0.05	-0.03	-0.04	0.00	-0.01	-0.02	-0.03	-0.01	-0.01	-0.02	-0.02	-0.03	-0.01	0.04	-0.02	-0.03	-0.05	-0.02	0.08	0.17	0.21
**ION 318**	-0.02	-0.02	-0.03	-0.04	-0.02	0.01	-0.01	-0.01	-0.02	0.00	0.00	-0.02	0.00	-0.03	-0.01	0.02	-0.03	-0.02	-0.03	0.00	0.00	0.31	0.31
ION_354	-0.04	-0.03	-0.06	-0.04	-0.04	-0.01	-0.01	0.06	-0.04	-0.03	-0.02	-0.04	-0.04	-0.04	-0.01	0.02	-0.01	-0.04	-0.06	0.12	0.21	0.35	0.40
ION_324	-0.01	0.00	-0.02	0.00	-0.02	0.01	0.00	-0.01	-0.01	0.00	0.00	0.01	-0.01	-0.02	0.02	0.07	0.02	-0.01	-0.02	0.15	0.01	0.13	0.13
**ION 327**	-0.01	0.00	-0.02	-0.01	-0.02	0.01	0.00	0.00	-0.01	0.00	-0.01	0.00	-0.01	-0.03	0.01	0.06	0.01	-0.01	-0.02	0.13	0.00	0.09	0.08
**ION 363**	0.01	0.00	-0.06	-0.03	-0.03	-0.01	0.00	0.02	-0.03	-0.02	-0.02	-0.03	0.00	-0.02	-0.01	0.01	0.06	0.47	0.10	-0.03	0.18	0.37	0.30
CoV2-2130	-0.01	-0.02	-0.04	0.25	0.26	0.28	0.26	0.22	0.22	0.26	0.18	0.23	0.12	0.26	0.26	0.18	0.25	-0.02	-0.03	0.24	-0.01	0.24	0.25
ION_300	0.62	0.59	0.53	0.57	0.64	0.66	0.60	0.45	0.50	0.57	0.40	0.51	0.71	0.64	0.62	0.70	0.53	0.63	0.62	0.53	0.29	-0.01	-0.01
ION_321	0.44	0.48	0.38	0.51	0.57	0.61	0.51	0.38	0.41	0.51	0.35	0.45	0.61	0.58	0.53	0.61	0.41	0.55	0.19	0.41	0.15	-0.03	-0.03

Figure S4. Epitope binning with RBD on panel of 21 antibodies and controls. A) Assay format. Using Octet BLI technology, in both orientations (i.e. Capture vs Detector) a classical sandwich assay format was employed to identify the presence of seven different epitope bins. Two characterized control antibodies (COV2-2130 and COV2-2196) were also included in the analyses. B) Epitope binning results matrix. All antibodies were tested as Ab1 – Capture and as Ab2 – Detector. The signals are organized in a matrix, where the self-self pairing responses are displayed along the diagonal. Responses above 0.1nm were considered as positive (i.e. presence of viable pairing).

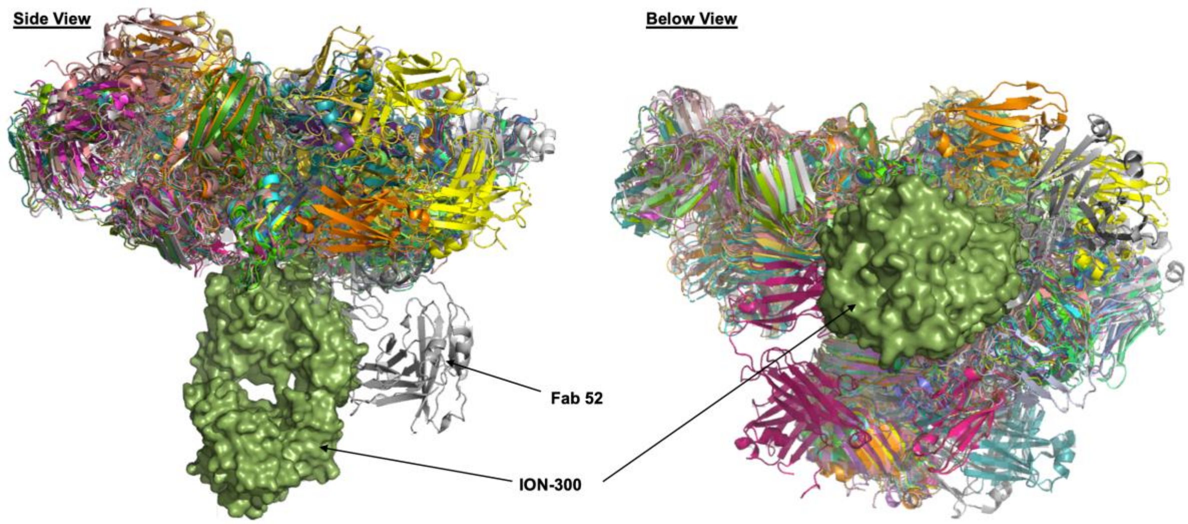

Figure S5. Superposition of antibody and nanobody structures targeting the SARS-CoV-2 RBD. Cartoon representation of all published antibody and nanobody structures that bind to the SARS-CoV-2 RBD, superposed onto the RBD of the ION_300: RBD structure (green molecular surface), from the side and below viewpoints of the ION_300 molecule.

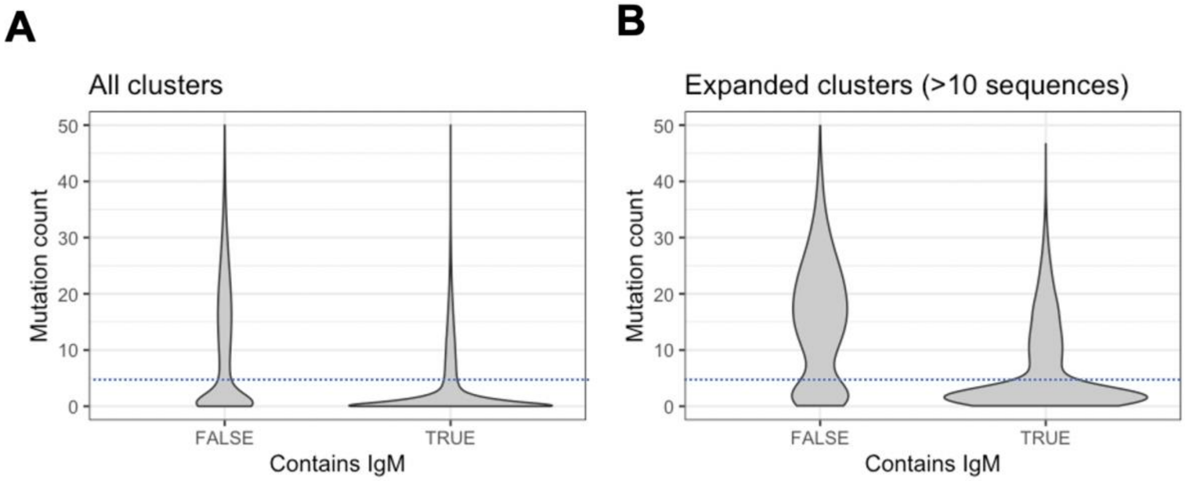

Figure S6. Mutation distribution of clusters. A) Clusters were stratified according to whether they contained any IgM sequences. Violin plot shows the average mutation of the clusters within each group. B) Same as A, but only including clusters that contain >10 sequences. For A and B, the dotted line indicates the cutoff that was used to distinguish naïve, or recently activated cells from memory cells.

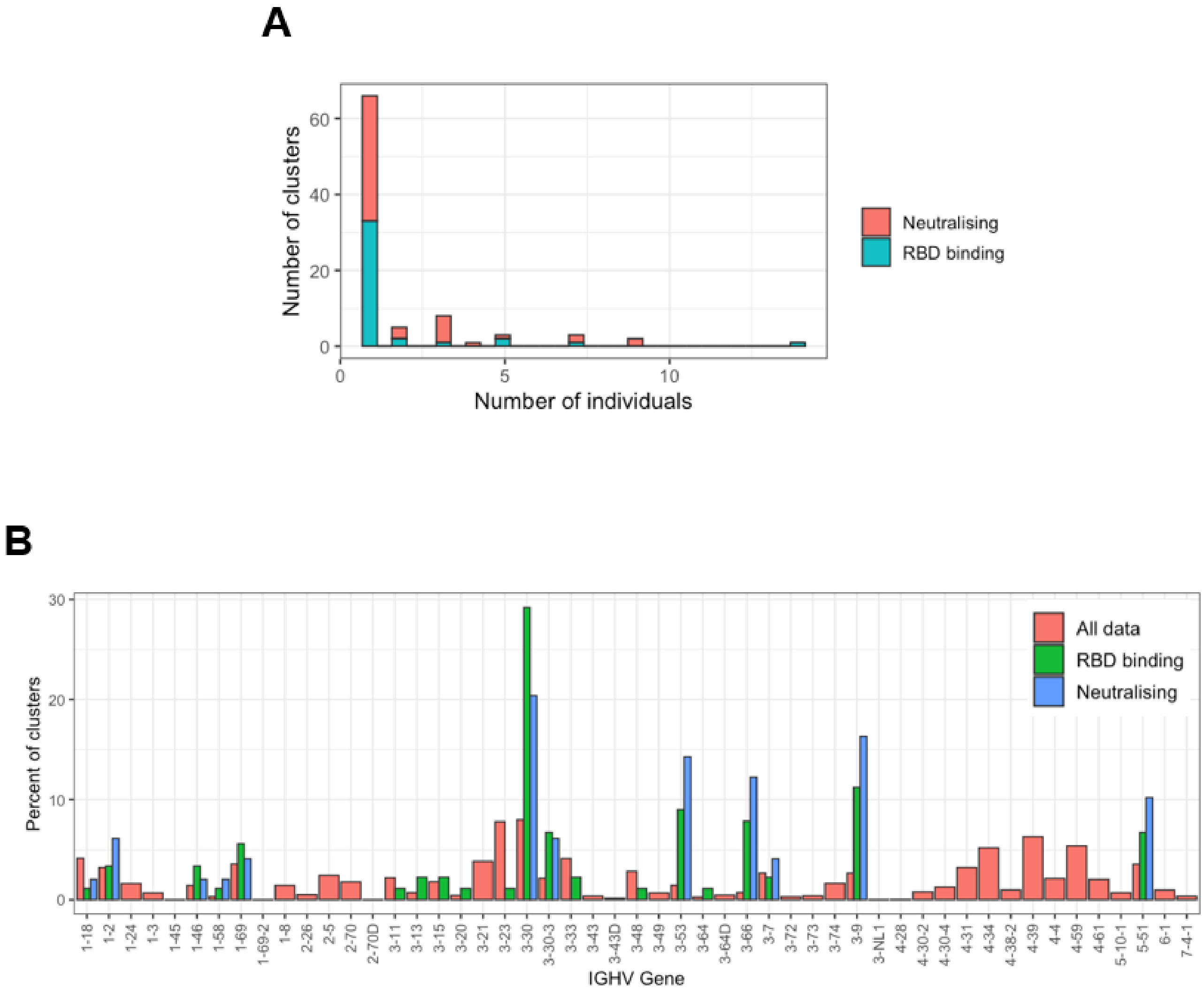

Figure S7. VH germline usage in convergent antibody response. A) The number of individuals the clusters annotated as RBD-binding or RBD binding and neutralizing are present in. B) V gene segment usage distribution of clusters.

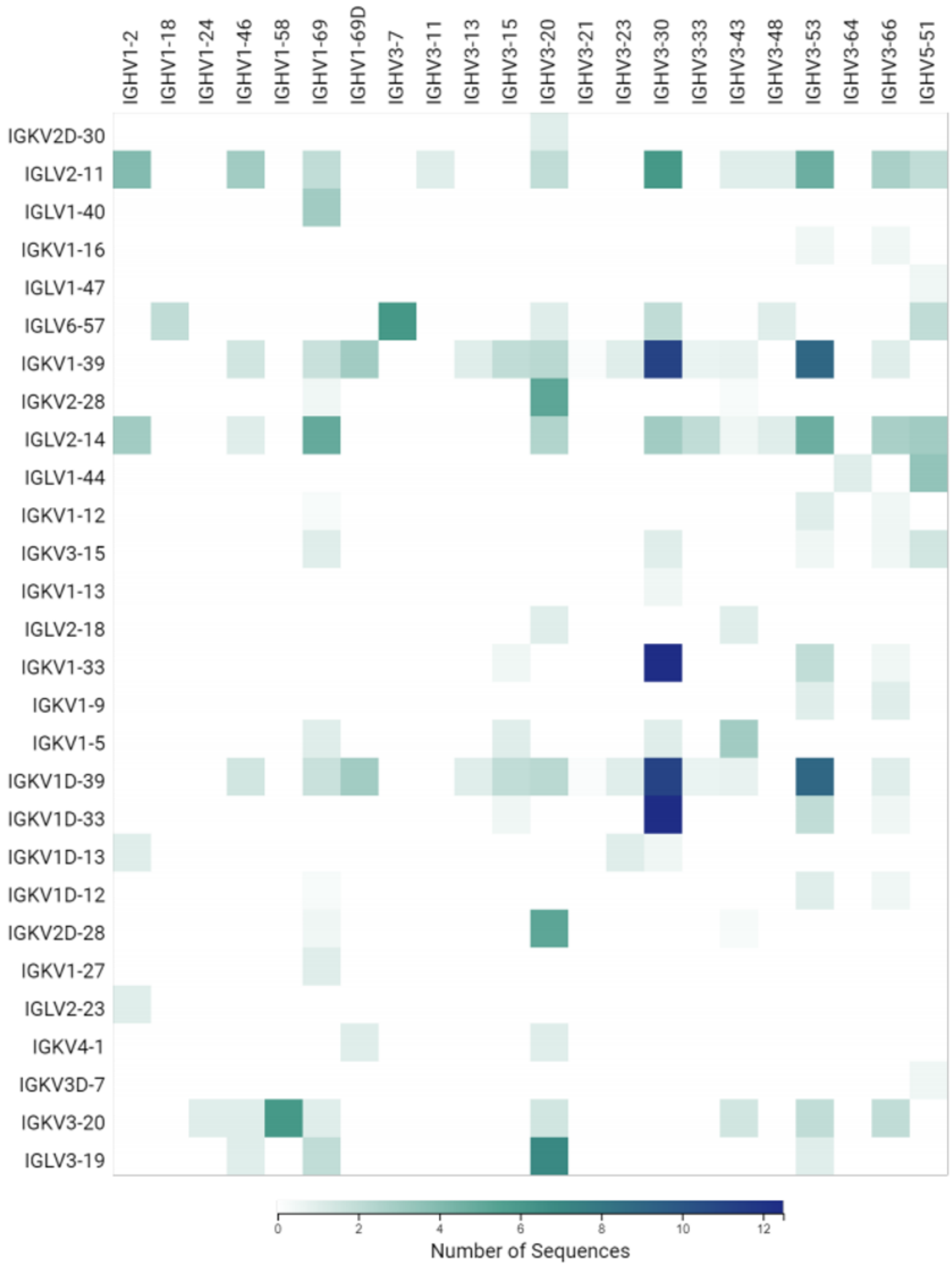

Figure S8. Heavy and light chain V gene utilization and pairing preference of all RBD-binding antibodies isolated using phage display technology. Analysis performed with Geneious Biologics (Biomatters).

Reference	No. of days of symptoms (mean) prior to sample collection
Rogers et al[11]	17
Hansen et al[5]	24.5
Zost et al[a][7]	50
Cao et al[6]	38
Zost et al[8]	43
Liu et al[52]	25
Kreer et al[29]	25
Brouwer et al[31]	28
Bin Ju et al[53]	17
Robbiani et al[27]	39
Tortorici et al[54]	53.5
Mean	32.7

Table S1. Days of symptoms prior to sample collection in related studies.

Donor ID	Age group	Gender	Ethnicity	No. of days of symptoms prior to sample collection	Clinical status
BARTS_01	30-39	Female	Caucasian	7	Deteriorating
BARTS_02	30-39	Male	SE Asian	10	Stable
BARTS_03	50-59	Male	Caucasian	11	Stable
BARTS_04	70-79	Male	Black	14	Stable
BARTS_05	70-79	Female	SE Asian	7	Stable
BARTS_06	30-39	Male	N/A	14	Improving
BARTS_07	30-39	Male	N/A	4	Improving
BARTS_08	50-59	Male	SE Asian	15	Stable
BARTS_10	70-79	Female	Caucasian	10	Stable
BARTS_11	20-29	Male	SE Asian	9	Stable
BARTS_12	80-89	Female	Caucasian	Uncertain	Stable
BARTS_13	20-29	Male	Asian	11	Stable
BARTS_14	40-49	Male	Caucasian	6	Deteriorating
BARTS_15	30-39	Male	SE Asian	11	Stable
BARTS_16	50-59	Female	N/A	11	Stable
BARTS_17	40-49	Female	Black	12	Stable
BARTS_18	30-39	Male	Caucasian	20	Improving
BARTS_19	40-49	Male	SE Asian	15	Improving

Table S2. Clinical details and characteristics of the patient cohort used in this study.
In this cohort, 12 (66.7%) patients were male and 6 were females (33.3%), and had a mean age of 48.8 (range 25.6-87.4) years. The clinical status of the patient on the day of sample collection was subjectively assigned as Improving, Stable or Deteriorating by the direct clinical care team, on the basis of increasing, stable, or decreasing requirement of supplemental oxygen in comparison to the previous three days.

Antibody ID	K_{on} [$M^{-1}s^{-1}$]	K_{off} (s^{-1})	KD [nM]
ION_300	2.52×10^{5}	7.75×10^{-4}	3.4
ION_303	9.93×10^{5}	4.60×10^{-3}	4.8
ION_309	5.57×10^{5}	4.27×10^{-3}	7.8
ION_312	1.18×10^{6}	1.44×10^{-2}	12.4
ION_315	6.73×10^{5}	1.14×10^{-2}	17.0
ION_318	6.97×10^{5}	4.70×10^{-3}	6.8
ION_321	4.26×10^{5}	2.73×10^{-4}	0.7
ION_324	1.00×10^{6}	2.99×10^{-2}	31.9
ION_327	9.86×10^{5}	4.20×10^{-2}	44.0
ION_330	3.27×10^{5}	8.82×10^{-3}	27.4
ION_333	2.38×10^{5}	4.11×10^{-3}	17.3
ION_336	8.03×10^{4}	1.22×10^{-3}	37.5
ION_339	3.85×10^{5}	1.71×10^{-3}	4.5
ION_342	3.12×10^{5}	7.65×10^{-3}	24.6
ION_345	1.76×10^{5}	3.71×10^{-3}	22.2
ION_348	5.79×10^{5}	3.12×10^{-2}	55.7
ION_351	1.86×10^{5}	2.91×10^{-4}	1.6
ION_354	1.73×10^{5}	1.23×10^{-3}	7.1
ION_357	1.09×10^{5}	2.42×10^{-4}	2.7
ION_360	1.88×10^{5}	2.79×10^{-4}	1.5
ION_363	6.12×10^{5}	4.39×10^{-5}	0.07

Table S3. 1:1: binding kinetics of the final panel of 21 antibodies to SARS-CoV-2 RBD measured by SPR.

Antibody ID	Pseudovirus neutralization IC_{50} in nM (SEM)	Authentic virus neutralization IC_{50} in nM (SEM)
ION_300	>5	16.00 (1.74)
ION_303	0.107 (0.011)	0.14 (0.06)
ION_309	0.044 (0.047)	0.37 (0.06)
ION_312	0.086 (0.022)	0.14 (0.06)
ION_315	0.064 (0.011)	0.28 (0.08)
ION_318	0.048 (0.007)	0.10 (0.02)
ION_321	>5	4.15 (2.93)
ION_324	0.031 (0.022)	0.24 (0.04)
ION_327	0.116 (0.018)	0.24 (0.01)
ION_330	0.041 (0.014)	0.17 (0.06)
ION_333	0.043 (0.007)	0.12 (0.03)
ION_336	0.083 (0.028)	1.01 (0.09)
ION_339	0.052 (0.041)	0.40 (0.05)
ION_342	0.064 (0.000)	0.51 (0.09)
ION_345	0.073 (0.018)	0.25 (0.05)
ION_348	1.181 (0.542)	3.20 (0.20)
ION_351	0.061 (0.010)	0.24 (0.004)
ION_354	0.059 (0.011)	0.19 (0.03)
ION_357	0.183 (0.029)	0.69 (0.10)
ION_360	0.042 (0.005)	0.35 (0.03)
ION_363	0.142 (0.024)	0.29 (0.05)
COV2-2130	0.099 (0.020)	0.84 (0.36)
COV2-2196	0.016 (0.000)	0.12 (0.03)
S309	1.417 (poor fit)	2.40 (0.84)

Table S4. Neutralization potency of the selected 21 antibodies against a pseudotyped virus. Neutralizing antibody titre is expressed as 50% inhibitory concentration (IC_{50}). IC50s were calculated from at least two independent experiments conducted in triplicates. Control antibodies COV2-2130, COV-2196 and S309 were also included.

	RBD:ION_300	RBD:ION_360
Data Collection		
Beamline	ID30A1	ID30A1
Wavelength (Å)	0.96861	0.97950
Space Group	$P4_3$	$P2_1$
Cell Dimensions		
a, b, c (Å), α, β, γ (°)	75, 75, 143, 90, 90, 90	91, 108, 182, 90, 99, 90
Resolution (Å)	40.23 – 2.35	48.28 – 2.80
R_{merge}	0.034 (0.605)	0.074 (0.553)
$CC_{1/2}$	0.997 (0.480)	0.990 (0.491)
I/σI	16.3 (1.5)	5.8 (1.3)
Completeness (%)	99.1 (99.9)	99.9 (99.1)
Redundancy	1.9 (1.8)	2.0 (2.0)
Refinement		
No. of Reflections	61133 (5967)	170510 (16939)
No. of Unique	32467 (3234)	85424 (8446)
R_{factor} / R_{free} (%)	22.4 (26.5)	23.2 (28.1)
Wilson *B*-factors (Å)	56.5	66.6
B-factors (Å)		
Protein	70.3	64.9
R.M.S. Deviations		
Bond lengths (Å)	0.004	0.004
Bond angles (°)	0.830	0.810
Ramachandran Favoured (%)	95.2	94.5

Table S5. X-ray data and refinement statistics.

Convergent cluster ID	BARTS_01	BARTS_02	BARTS_03	BARTS_04	BARTS_05	BARTS_06	BARTS_07	BARTS_08	BARTS_10	BARTS_11	BARTS_12	BARTS_13	BARTS_14	BARTS_15	BARTS_16	BARTS_17	BARTS_18	BARTS_19
1	0	223	5	0	26	0	72	0	0	37	0	1	0	83	0	6	0	1227
2	0	0	2	2	0	81	1	0	0	29	0	0	7	36	4	0	2	0
3	0	57	1	3	88	58	11	4	2	172	0	0	2	306	5	0	4	489
4	0	3	0	0	0	0	0	26	0	0	0	0	0	38	0	0	0	2
5	0	0	0	0	0	36	6	0	0	0	0	0	0	0	0	0	1	0
6	0	22	0	0	0	0	1	0	0	0	0	0	0	22	0	0	0	0
7	0	44	0	0	0	0	0	0	0	0	0	2	0	0	0	0	0	26

Table S6. Convergent clusters across individuals. The number of times each convergent cluster (highlighted in Table 3.1) appeared within each patient donor (numbered BARTS_01 to BARTS_19) is shown.
